# A Framework for a Statistical Characterization of Epidemic Cycles: COVID-19 Case Study

**DOI:** 10.2196/22617

**Published:** 2021-03-18

**Authors:** Eduardo Atem De Carvalho, Rogerio Atem De Carvalho

**Affiliations:** 1 Center for Science and Technology Universidade Estadual do Norte Fluminense Campos Brazil; 2 Innovation Hub Instituto Federal Fluminense Campos Brazil

**Keywords:** COVID-19, SARS-CoV-2, pandemics, infection control, models, experimental, longitudinal studies, statistical modeling, epidemic cycles

## Abstract

**Background:**

Since the beginning of the COVID-19 pandemic, researchers and health authorities have sought to identify the different parameters that drive its local transmission cycles to make better decisions regarding prevention and control measures. Different modeling approaches have been proposed in an attempt to predict the behavior of these local cycles.

**Objective:**

This paper presents a framework to characterize the different variables that drive the local, or epidemic, cycles of the COVID-19 pandemic, in order to provide a set of relatively simple, yet efficient, statistical tools to be used by local health authorities to support decision making.

**Methods:**

Virtually closed cycles were compared to cycles in progress from different locations that present similar patterns in the figures that describe them. With the aim to compare populations of different sizes at different periods of time and locations, the cycles were normalized, allowing an analysis based on the core behavior of the numerical series. A model for the reproduction number was derived from the experimental data, and its performance was presented, including the effect of subnotification (ie, underreporting). A variation of the logistic model was used together with an innovative inventory model to calculate the actual number of infected persons, analyze the incubation period, and determine the actual onset of local epidemic cycles.

**Results:**

The similarities among cycles were demonstrated. A pattern between the cycles studied, which took on a triangular shape, was identified and used to make predictions about the duration of future cycles. Analyses on effective reproduction number (R_t_) and subnotification effects for Germany, Italy, and Sweden were presented to show the performance of the framework introduced here. After comparing data from the three countries, it was possible to determine the probable dates of the actual onset of the epidemic cycles for each country, the typical duration of the incubation period for the disease, and the total number of infected persons during each cycle. In general terms, a probable average incubation time of 5 days was found, and the method used here was able to estimate the end of the cycles up to 34 days in advance, while demonstrating that the impact of the subnotification level (ie, error) on the effective reproduction number was <5%.

**Conclusions:**

It was demonstrated that, with relatively simple mathematical tools, it is possible to obtain a reliable understanding of the behavior of COVID-19 local epidemic cycles, by introducing an integrated framework for identifying cycle patterns and calculating the variables that drive it, namely: the R_t_, the subnotification effects on estimations, the most probable actual cycles start dates, the total number of infected, and the most likely incubation period for SARS-CoV-2.

## Introduction

The analysis of the life cycles of any epidemic involves the analysis of a series of quantitative parameters that govern these cycles and which, given the inherent uncertainty of these events, are generally treated by statistical models. For a number of practical reasons, the registration of deaths and of infections are inevitably imprecise, although these numbers can be corrected over time. Therefore, with the COVID-19 pandemic, a subject that immediately became the center of debates and different studies was the characterization of the different local epidemic cycles and their corresponding variables. Local cycles are those that have occurred or occur in specific countries, regions, or cities, and not the pandemic cycle as a whole, as the virus does not spread instantly across continents. Thus, it can be seen that some countries were in more advanced epidemic stages than others whose first infections were detected later. In other words, as expected, different “infection windows” coexist in parallel in different locations, with some locations at a more advanced stage, while others present more “delayed” cycles. Thus, numerically analyzing the behavior of early cycles was the measure undertaken by a series of researchers.

Although it is not the only one, as will be seen in this paper, the reproduction number is considered the central variable in the analysis of epidemic cycles. In order to determine the reproduction number, different categories of models have been proposed: artificial neural networks [1], Poisson [2,3], exponential [4], Markov chain [5], Gaussian [6,7], Weibull [8], Logistic-S [9], and moving averages [10]. Most research tries to frame the local epidemic cycles into Gaussian and/or Weibull behaviors, creating complex models that still led to errors in predictions, as we now know. More importantly, Park et al [11] showed that the initial models, most based on the Gaussian distribution and its derivatives, failed to make their predictions. After observing these findings, we saw that there was room to propose a framework that would provide an efficient and more comprehensive analysis of the epidemic cycles, going beyond the calculation of the reproduction number. Moreover, it would be both easy to understand and to compute, since local authorities, especially in low-income countries, do not always have statistical experts at their disposal to propose, calibrate, and analyze the results of complex models. Thus, based on experimental and publicly available data, we produced a series of studies that initially dealt with the identification of patterns in epidemic cycles and their use for predicting deaths [12], time-dependent effective reproduction number (R_t_) and subnotification effect estimation modeling [13], and finally, estimation of the actual onset of local epidemic cycles, determination of the total number of infected, and the duration of the incubation period [14]. In this paper, these findings are integrated and summarized in a coherent framework.

## Methods

Based on experimental data, the framework proposed here is divided into four parts: (1) applying the moving averages method and identifying the parameters of the epidemic cycle patterns, which are used to predict the number of future deaths in local epidemics, (2) modeling the R_t_ and (3) the effects of subnotification, and (4) applying the logistic model associated to a novel inventory model to obtain the final count for the total infected, the daily infection rate and lag time, and the incubation period.

### Patterns of Epidemic Cycles

Our method began with the observation of several cycles in western countries where the pandemic hit earlier, especially in Europe. From there, patterns were identified and predictions were applied. The attempt to describe the different epidemic cycles that make up the current pandemic often comes up against the quality of the data that is made public. Most data made public are based on “date of recording,” which is different of “day of death,” meaning that the date that a given set of deaths are recorded in the public health statistics systems is not necessarily the date they occurred on; given the usual bureaucratic procedures, recording may be delayed.

The fact is that the distribution of fatalities suffers a distortion that generates a “saw” appearance in graphs such that on weekends there is a clear absence of death records, followed by an explosion of values at the beginning of the week. A simple technique that softens this effect is to apply the so-called moving average method (MAM), in which the daily value of deaths is replaced by the sum of the previous 6 days with the current day, divided by 7; in other words, the average of the week ended in the current day. In particular, MAMI (MAM with initial value) will be used here, which entails assigning the average of the 7 days to the first day of the week (Sunday).

In the period in which the data were obtained and analyzed (first week of July 2020), several cities, regions, states, and countries had already completed what will be called here the most lethal cycle of the epidemic (MLCE), which is when the number of deaths increases daily, on average, until it reaches a peak and then begins to decrease continuously until it reaches a minimum value. After this period, the occurrence of deaths continues intermittently, but relatively small and oscillating, decreasing to certain levels of daily deaths, where it then becomes apparently chronic and presents relatively low values, but remains greater than zero.

In order to show numerical cases of the application of the proposed model, data from three European countries with different cycles were analyzed: Germany, a country that was reported as exemplary in terms of application of nonpharmaceutical interventions (NPIs); Italy, which stayed at the center of the initial crisis; and Sweden, which generally did not apply any strong NPIs. The data for this part of the study were obtained from the Worldometer’s COVID-19 portal [15] as of July 9, 2020, and is presented, together with the calculations, in Multimedia Appendix 1.

#### Germany

**Figure 1:**
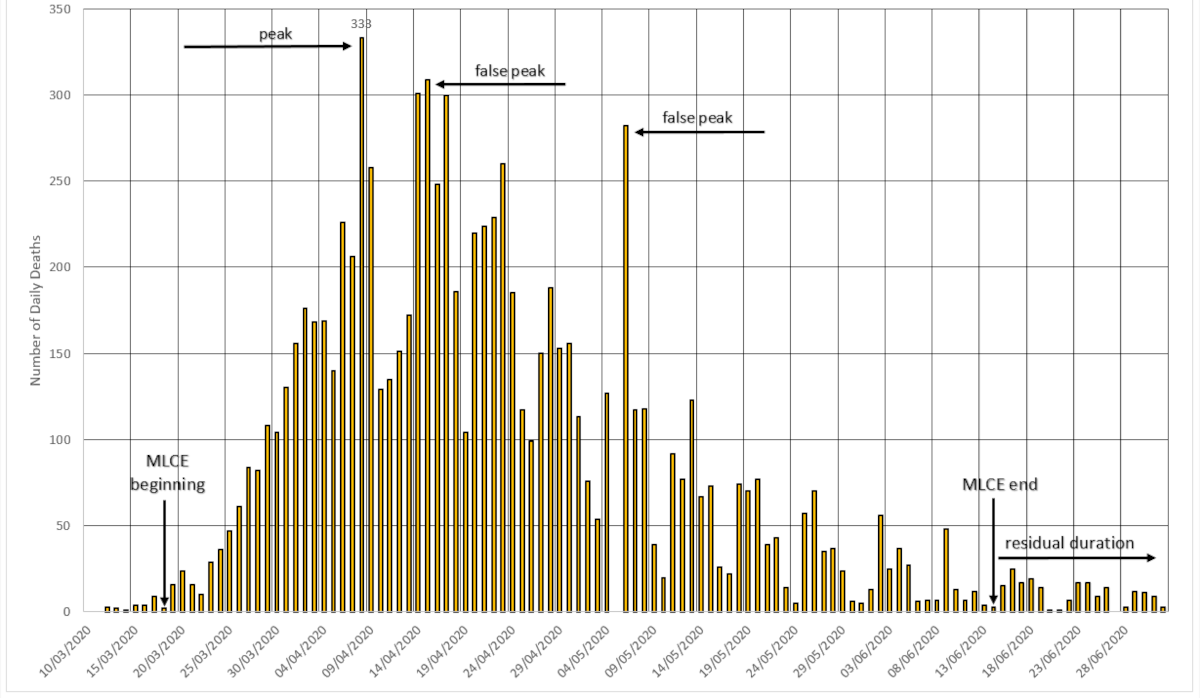
The cycle in Germany. MLCE: most lethal cycle of the epidemic. Source: Worldometer [15].

Described from the beginning of the pandemic as a country that managed the crisis in an exemplary way, testing significant portions of its population and controlling and lifting restrictions on public movement based on well-known numbers and percentages of cases. Figure 1 shows the evolution of deaths in Germany. This framework points to the existence of the so-called false peaks. These are local maximums that were recorded during the cycle of rising or falling in the trend of deaths, but they are not inflection points. In order for a point to be considered as a (real) peak, it is necessary to register a tendency of decline in the number of deaths. This fall will not be linear, but there is an obvious, numerical, and visual trend that indicates such a pattern.

#### Italy

**Figure 2:**
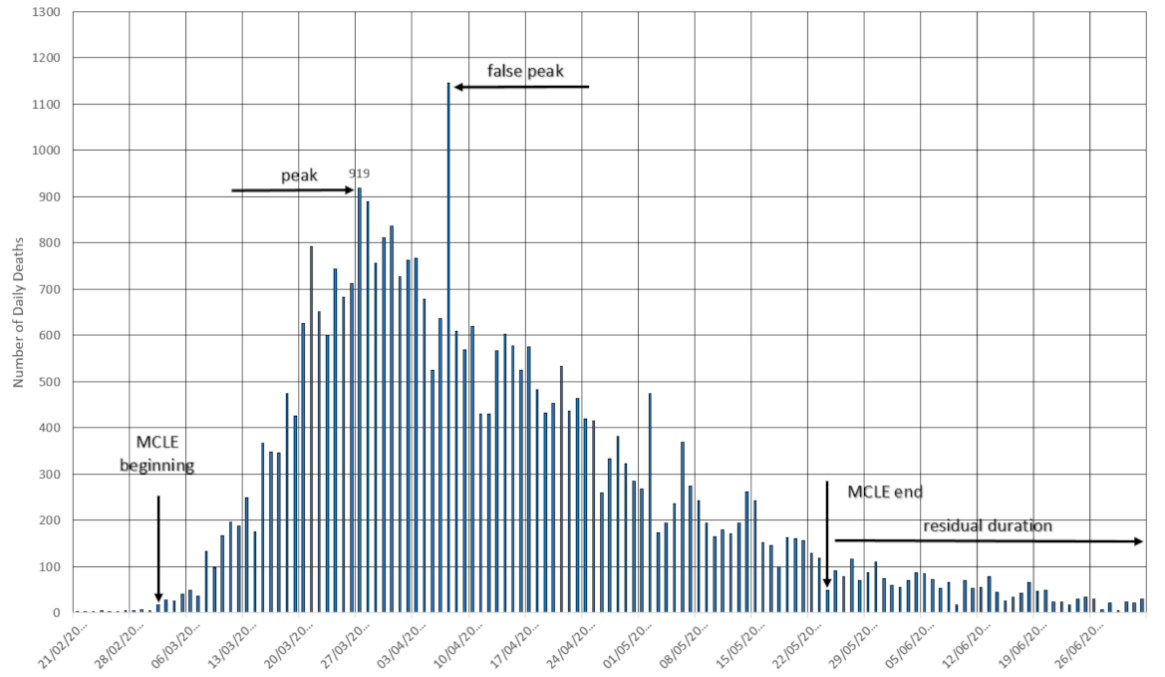
The cycle in Italy. MLCE: most lethal cycle of the epidemic. Source: Worldometer [15].

A country that was at the European epicenter of the crisis, Italy experienced an evolution in the number of deaths (Figure 2), which indicates the overcoming of the MLCE.

#### Sweden

**Figure 3:**
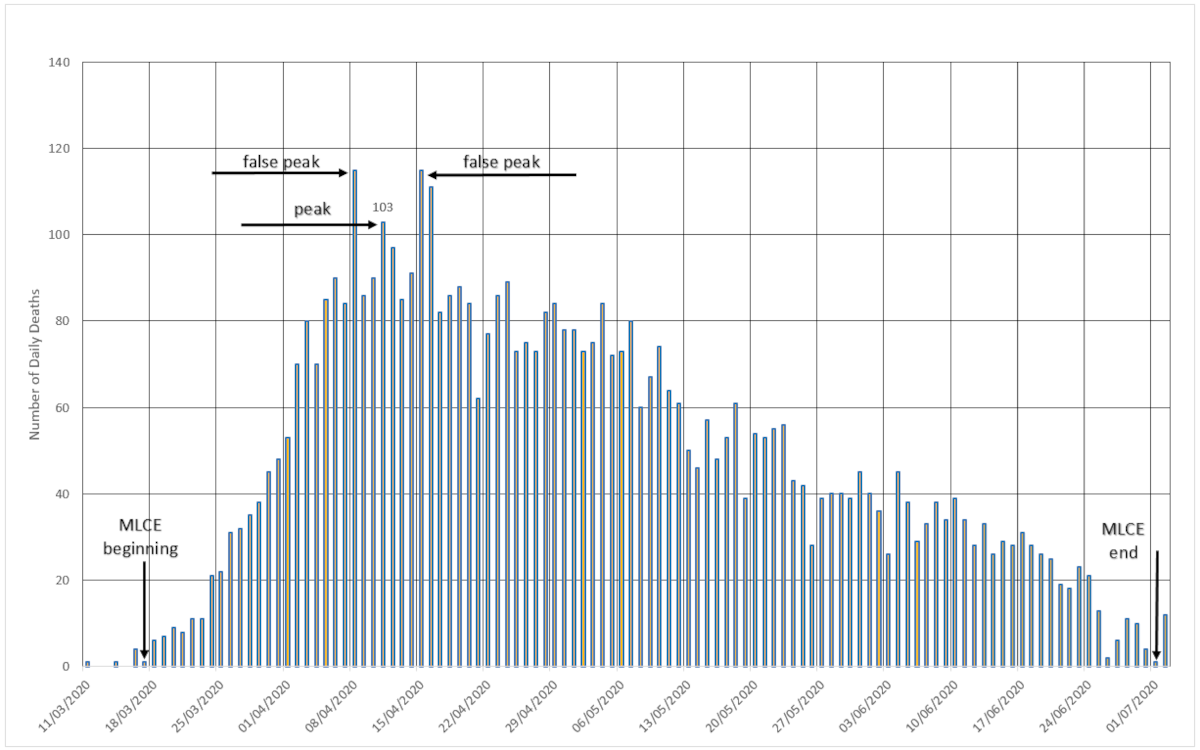
The cycle in Sweden. MLCE: most lethal cycle of the epidemic. Source: Worldometer [15].

Sweden, an European country that has not adopted the practices of radical social isolation like its neighbors, has a cycle of aspect not unlike that of all other European countries. Figure 3 shows the values of deaths that have already been corrected for the dates on which they actually occurred and not the date of registration.

### Nondimensional Characteristics of Epidemic Cycles

**Figure 4:**
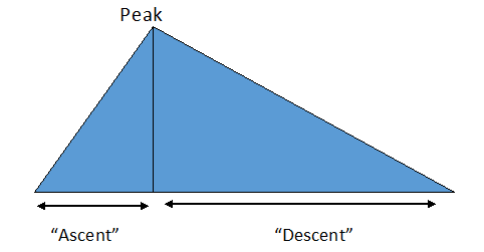
The generic shape of COVID-19 lethal cycles.

In general, the epidemic cycles described here have some common geometric characteristics, the main one being a triangular aspect (Figure 4), where a smaller side is formed, which corresponds to an average daily increase in the number of deaths until a peak is reached. This peak may be easily identifiable or require extrapolation of a line because the values oscillate naturally and some spurious points (false peaks) may appear. The peak is followed by a period where the number of deaths occurring daily tends to decrease on average. This period, for the observed cases, is longer than the previous one. According to Kotz and Rene van Dorp [16], the triangular distribution is used when there is no exact idea of what the distribution is, although there is an idea of the minimum and maximum values for the variable. Therefore, this distribution was chosen given its particular nature and use in situations where the description of a given population is uncertain, as is in this case. This distribution is based on the minimum and maximum estimates. Hence, Table 1 gathers values of the so-called triangular cycles presented earlier.

The values listed in Table 1 indicate that the period of rise of the disease in countries of relatively small sizes or in big cities is about 21 days, ranging from 19 to 25 days before reaching the so-called peak. From then until the end of this critical period, about 60 days pass, ranging from 45 to 81 days. The ratio between the two periods oscillates between 2.1 and 3.3, with an average of 2.8. Table 2 shows the number of deaths in the periods described above.

**Table 1:** Proportions between the time of ascent until the peak of deaths and descent to the end of the most severe cycle of COVID-19.

Country	Start	Peak	End	Days to the peak	Days to the end	Proportion between ascent and descent
Italy	March 7	March 27	May 24	20	57	2.9
Sweden	March 17	April 11	July 1	25	81	3.2
Germany	March 18	April 8	June 14	21	69	3.3

**Table 2:** Proportions between the number of deaths associated with the cycle of rising to the peak and of descending to the end of the most severe cycle of COVID-19.

Place	Start	Peak	End	Deaths to the peak	Deaths to the end	Proportion between ascent and descent
Italy	March 7	March 27	May 24	8937	24,082	2.7
Sweden	March 17	April 11	July 1	1255	4141	3.3
Germany	March 18	April 8	June 14	2323	6521	2.8

The values listed in Table 2 indicate that the number of deaths during the period of ascent of the disease in countries of relatively small sizes or cities is about 5791 (range 1255-10,293) before reaching the peak. From then until the end of this critical period, about 12,673 (range 4141-24,082) deaths occur. The ratio of death figures ranges from 1.6 to 3.3, with an average of 2.4.

Therefore, it is possible to identify that once the scale effects are removed, what remains is a spectrum of proportions of the epidemic cycle. Then, when submitting the data to the moving average method with the initial value (MAMI), there is a minimization of the effect of seasonality in the registration of deaths, caused by weekends, holidays, and other local peculiarities. After dividing all the values previously transformed by the peak of the series (peak now determined by MAMI), the values start to be dimensionless and fall between 0 and 1. In this way, the epidemic cycles can be compared with each other, since what remains are the proportions between the ascent, the peak, and the descent of the cycle. The time period does not change. One clear limitation of this method is the necessity of identifying the real peak. Then, a hypothesis arises that different locations may, under different behavioral rules, present the same behavior.

### Algorithm for Cycle Predictions

After identifying the triangular pattern and through successful application in several cases, a prediction algorithm was developed, described by the following steps:

1. MAMI is calculated for the daily figures on the number of deaths.
2. The set of values is normalized and MAMI is also applied on that.
3. A continuous curve is generated on a graph with the x axis as the number of consecutive days of the epidemic cycle and the y axis as the dimensionless range from 0 to 1 (some points, the false peaks, can go beyond this).
4. Among countries or localities, we seek those that have already ended their critical epidemic cycle (MLCE) and that are visually similar to the curve obtained in step 3, although obviously on a different scale, becoming the locality of reference.
5. MAMI is applied to the locality of reference.
6. Data of the locality of reference are normalized.
7. Repeat step 3 for the data of the locality of reference.
8. Considering that the cycle of the locality of reference is finished, it will be positioned previously on the graph, in relation to the place where it is desired to estimate the probable end date of the critical cycle. One should then numerically superimpose the peak of the case under study with the reference.
9. Once the superposition is made, always moving the reference case, an extrapolation can be made using the reference case as a guide to the value to be determined. As the scale of the case studied has not been changed, it is enough to consult what day it would be in the future to know the probable date.
10. If there is no similar case, you can eliminate the last days, as discussed above, and extrapolate directly from the values obtained in the public databases.

### Effective Reproduction Number

After identifying the similarities between cycles, the next step is to calculate the R_t_, which is done on the experimental behavior of the curve. First, however, it is necessary to understand the effect of MAMI on the reproduction number.

### MAMI Effect on Reproduction Numbers

**Figure 5:**
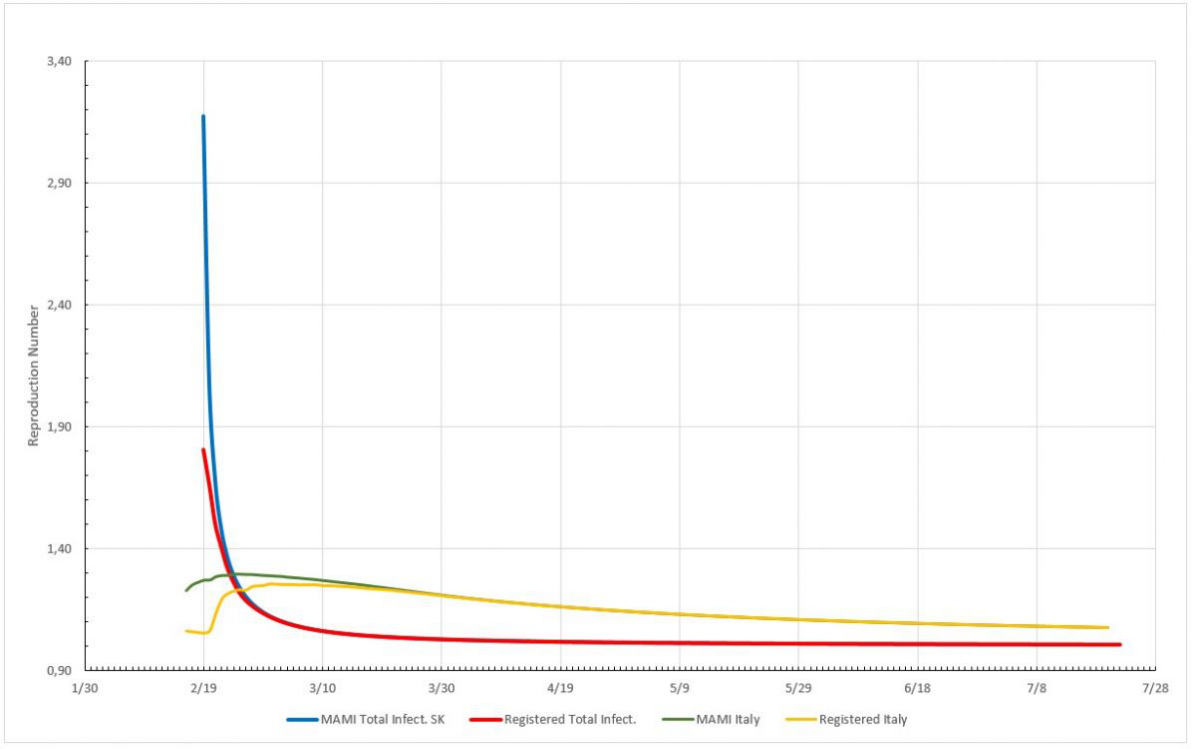
MAMI (moving average method–initial value) effect on reproduction numbers (R_t_) expressed for two different countries, South Korea (SK) and Italy. South Korea: the blue line is R_t_ obtained from MAMI applied to registered data; the red line is R_t_ determined for registered data. Italy: the yellow line is R_t_ for registered data; the green line is for MAMI applied to registered data. Source: Johns Hopkins University [17].

**Figure 6:**
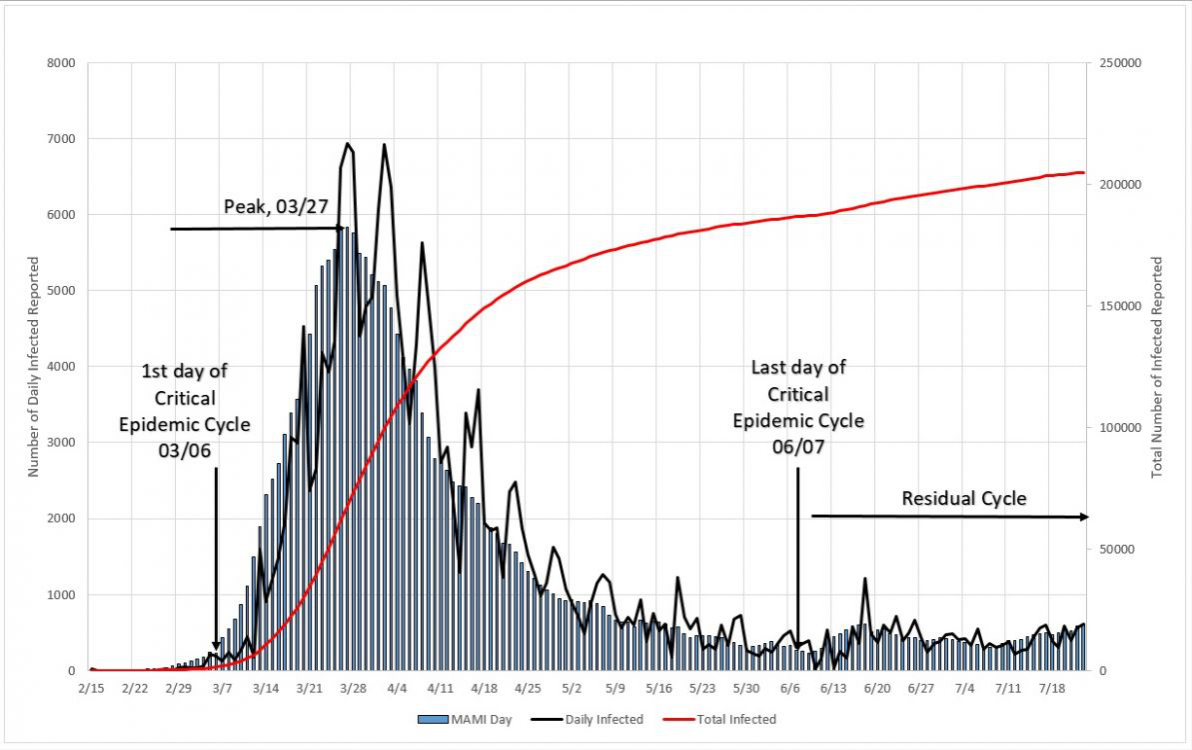
Number of COVID-19 cases reported for Germany. The black line represents the daily reported numbers, the blue bars their MAMI (moving average method–initial value), and the red line the total cases to date, using the right-hand axis as reference. Source: Johns Hopkins University [17].

**Figure 7:**
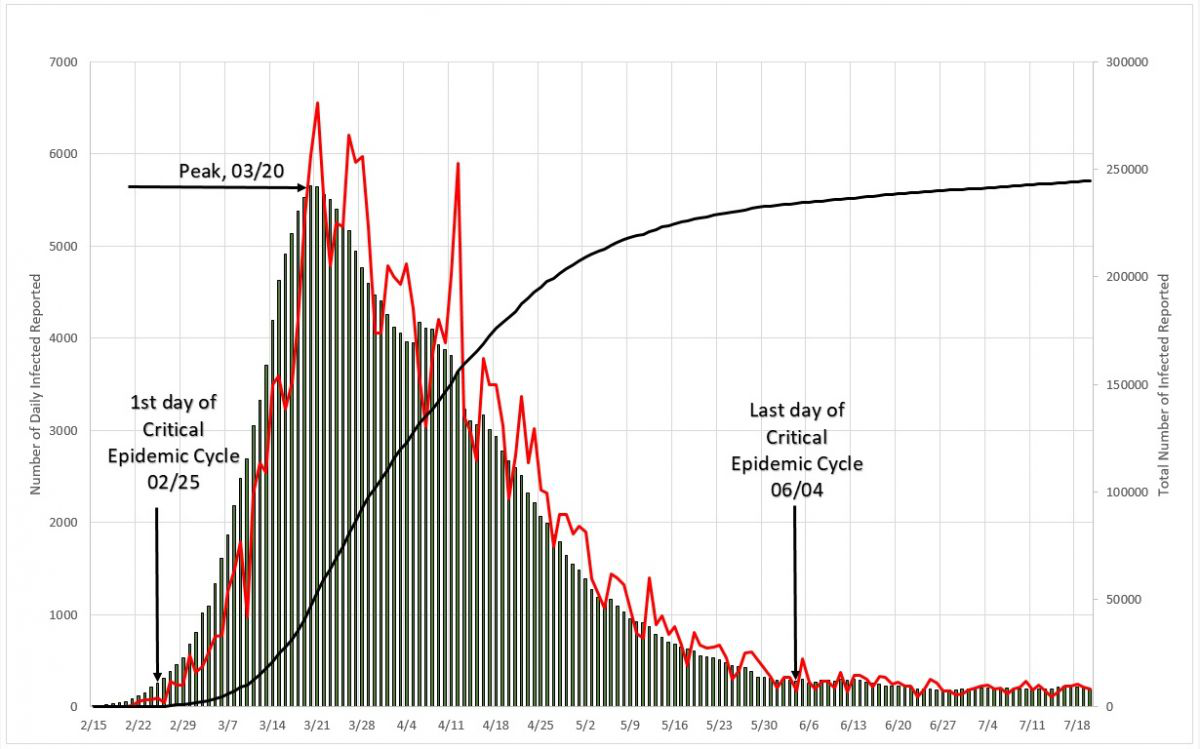
Number of COVID-19 cases reported for Italy. The black line represents the daily reported numbers, the blue bars their MAMI (moving average method–initial value), and the red line the total cases to date, using the right-hand axis as reference. Source: Johns Hopkins University [17].

**Figure 8:**
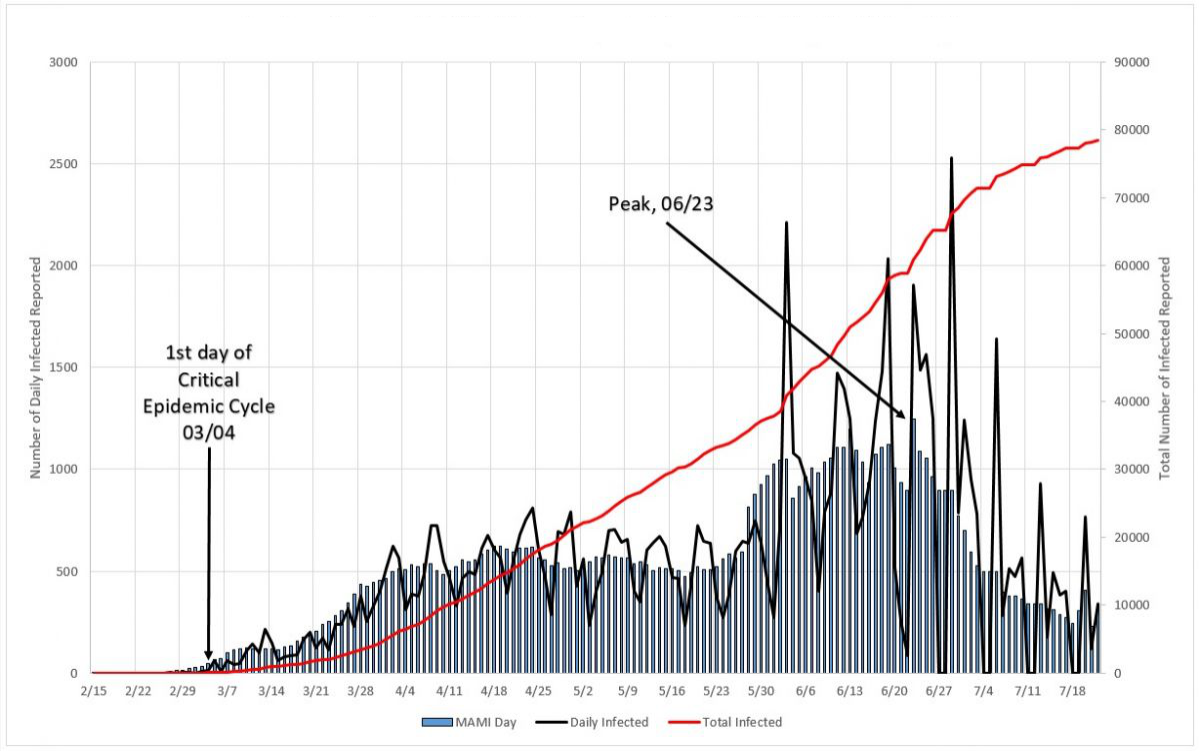
Number of COVID-19 cases reported for Sweden. The black line represents the daily reported numbers, the blue bars their MAMI (moving average method–initial value), and the red line the total cases to date, using the right-hand axis as reference.

The impact of MAMI applied to registered numbers can be better understood by analyzing Figure 5, where MAMI bears the greatest effect at the very beginning of the epidemic cycle; however, after a brief period, the average and actual data tend to yield to the same value as the cycles progress. It will be shown along this paper that the reproduction number varies most in the early stages, and the use of MAMI is plainly justified to avoid numbers that are registered in batches and not into a smooth daily fashion. Daily figures for total cases collected from the Johns Hopkins University’s website [17] on July 22, 2020, together with the calculations, are presented in Multimedia Appendix 2. The analysis of the R_t_ for the three European countries are represented in Figures 6-8.

### Deriving the Effective Reproduction Number

With the effect of moving averages measured, it is possible to proceed to an experimental method for calculating the daily number of infected and then an effective, time-varying reproduction number, calculating its value by means of experimental data outlined below.

The total number of infected daily (*I_d_*), during a period of time *t*, can be described as a function of the daily increase rate factor (1+*b*) multiplied by a scale factor, as shown in equation 1:

*I_d_=a (1+b)^t^***(1)**

In equation 1, *a* is the scale factor and *b* is the absolute daily increase rate, or instantaneous rate, and is defined as:


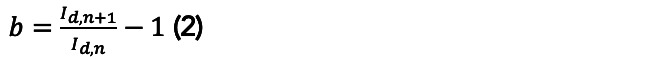


where *I_d,n+1_* is the current day and *I_d,n_* is the previous day.

Equation 1 can be written as:

*I_d_=C^t^***(3)**

where *C* is the time-dependent effective reproduction number, R_e_(t), or R_t_ for short, which is obtained from experimental data. For the reproduction number determination, it is necessary to determine the scale factor *a*. Therefore, *a* takes the following form:


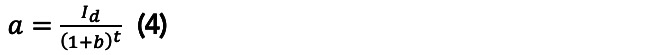


Finally, from equations 3 and 4:


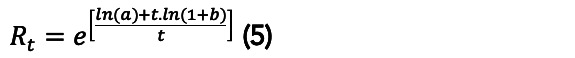


In order to map the interpretation proposed from equations 1 to 5 to the classical mathematical interpretation for the reproduction number (R_0_), an equivalence transformation will be described as follows. From the classical definition of R_0_, let:


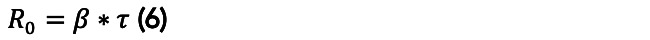


where β is infection-producing contacts per unit time (instantaneous rate), with a mean infectious period of τ. Equation 6 can be transformed into:

*R*_0_=*e^k^*^τ^**(7)**

From equations 5 and 7:


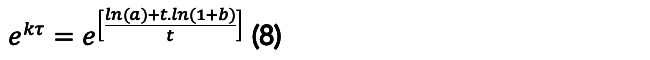


In equation 8, all dimensional units are compatible, therefore our transformations to obtain R_t_ in equation 5 are valid. Equation 5 was obtained from experimental data, and it is at the core of the model proposed here. From this point onward, R_t_ must be interpreted as R_e_(t) as explained before, in the interpretation of equation 3.

**Figure 9:**
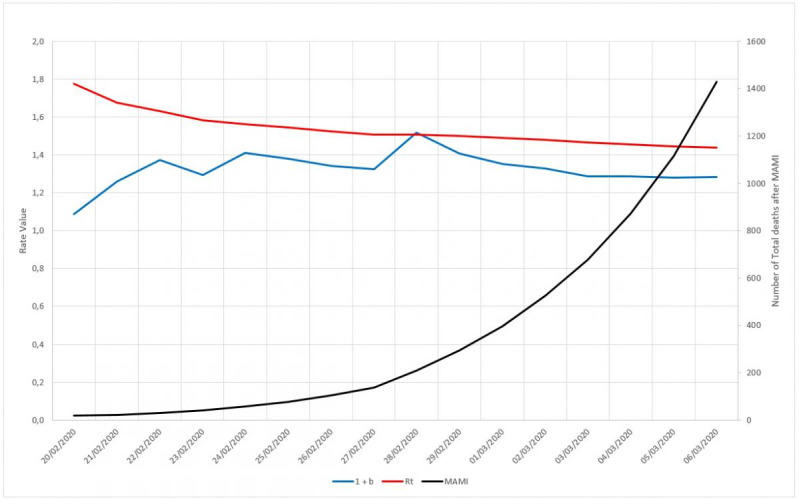
Behaviors of (1+b) and effective reproduction number (Rt) factors for the first 20 days in the epidemic cycle of Germany. MAMI: moving average method–initial value.

During the data analysis, we noted that the daily increase rate factor (1+*b*) is not enough to describe the number of contaminated cases registered in a given day, because it simply informs the absolute increase ratio that occurred from one day to the next. The reproduction number coefficient needs more numerical information in order to be able to express correctly the magnitude of daily numbers. It needs the scale factor *a* to bring more information on the phenomenon. As an example of this finding, Figure 9 shows that while the (1+*b*) factor varies rapidly, R_t_ drops steadily, changing slowly as the exponential time grows. The same behavior is displayed by the total daily registered number of deaths, which keeps growing smoothly. This is the numerical evidence that the factor (1+*b*) alone cannot describe the total number of deaths.

### Subnotification Effect on the Reproduction Number

When it comes to analyzing the number of cases of infection in the COVID-19 epidemic, an issue that always arises is underreporting or subnotification and its importance in predicting the behavior of the epidemic cycle. Thus, the third part of the framework is dedicated to the study of subnotification and its effects on prediction. Subnotification is understood as the fact that counts of infected persons are only estimated by public health authorities. Given that many people exposed to the virus do not display any sign of infection or the symptoms are very mild, therefore going unnoticed and unregistered by local bureaus of health statistics, the development of evaluation tools of the impact of these nonnotified cases is necessary. If it is assumed that subnotification is a constant factor (eg, 10 times the registered number of cases) during the whole epidemic cycle, it does not change the absolute daily increase rate *b* or the (1+*b*) factor. However, it does affect the scale factor *a*, therefore changing R_t_.

### Subnotification Impact Estimation Method

The impact of subnotification on R_t_ may be estimated by initially assuming that the actual registered figures for daily infected persons are no longer their actual values, but “real” ones multiplied by a factor—the subnotification factor. After that, the scale factor *a* is calculated. The term (1+*b*) remains constant, once the ratio (equation 3) remains constant. Then *a* and (1+*b*) are applied to equation 5, thus recalculating R_t_, now reflecting the effect of the imposed subnotification factor. This new R_t_ value would have been the correct one, in case all subnotified cases were suddenly registered. The percentage difference between this new, recalculated R_t_ and the actual one provides an estimate for the impact of subnotification on the reproduction number for a given population. Therefore, multiplying the values for registered cases by a factor of 10 will not cause a tenfold increase in R_t_. The true impact must be therefore calculated as described. It is also observed that subnotification mostly affects the very beginning of the critical cycle. After a certain amount of time, errors drop to insignificant values, below 5%.

### Total Number of Infected, Daily Infection Rate, Lag Time, and Incubation Period

The fourth component of the framework is the application of the logistic model to estimate three parameters: the total count of infected individuals; the daily infection rate; and the lag, which defines when the cycle actually started. An innovative model, based on the concept of inventory formation, is used to determine a fourth parameter—the most likely incubation period for the virus.

Considered by many authors as a good fit for modeling epidemic episodes [18-20], the logistic model describes three typical phases for this type of episode: the slow start, the steady growth, and finally the asymptotic behavior of the end. There are several ways to implement this function, and this work will use the so-called Richard growth model to describe the accumulated number of infection cases. The generalized logistic function has the following form:


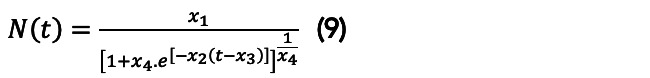


By selecting the highest *r*^2^ among several variations of equation 9, through curve-fitting, a particular form for equation 9 is:


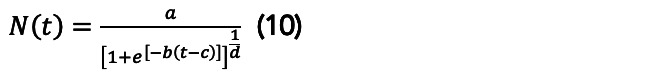


where *N(t)* is the number of infected persons at a given period of time *t*, *a* is the final count for the total infected, *b* is the daily infection rate, *c* is the lag phase, and *d* is a positive real number. It can be shown that:


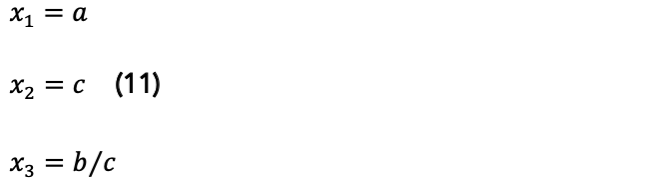


The constants *a*, *b*, *c*, and *d* will be used to estimate *x*_1_, the maximum number of infected people in a given location; *x*_2_ is the daily infection rate, or the average absolute daily increase in the number of infected, which can be used to determine the reproduction number (and to estimate the incubation period). Finally, *x*_3_ is used to estimate the lag time, or the actual moment when the first case occurred.

### Incubation Period Estimation

Although there is a series of studies on the incubation period for SARS-CoV-2, in order to maintain consistency within the framework, we sought to develop a model that could also estimate what would be the best incubation period estimation method to consider when modeling epidemic cycles. For that, we defined a model of inventory of infected people similar to the one used in productive systems, as shown in equation 12:

*I_t_* = *I_t–1_* + *D_t_* – *D_t–n_*** (12)**

where *I_t_* is the inventory of people infected in day *t*, or the total of infected in day *t*; *I_t-1_* is the inventory of people infected in the previous day; *D_t_* is the number of people detected with the disease in day *t*; and *D_t-n_* is the number of people detected with the disease *n* days before *t*.

Equation 12 should be interpreted as follows: the number of people who are infectious on a given day is equal to the number of people who were infectious the day before, plus the number of infected detected on the same day, and minus the number of people who have left the N-day incubation period. This reasoning therefore assumes that as soon as a person finds out he or she is infected, that is, when this person leaves the incubation period, enters perfect isolation and stops infecting. Although this assumption is not completely realistic—since it depends not only on individual responsibility, but also on the implementation of efficient isolation measures—at the same time it must also be considered that not every infected person effectively infects others, given that isolation is not the only way to avoid viral contamination. Thus, we consider this assumption to be reasonable enough to be applied statistically.

Other basic assumptions are that of all people susceptible (not vaccinated, sufficiently exposed to the pathogen, etc), not all will expose or develop the disease in a form severe enough to be noticed. Accordingly, the recorded number of daily cases does not reflect the total number of infected, but those who seek medical attention and therefore were diagnosed as contaminated. Hence, this is the number of infected in a given day, or the “inventory” of people that can infect other people in a given day. With the formulation defined in equation 12 and the assumptions described previously, we carried out the analysis and simulations for the three countries.

## Results

### General Findings

The epidemic cycles observed were subjected to the numerical methods present in the framework and described in the previous section. The first data transformation was the application of the MAMI value. The second transformation was normalization, where all the values were divided by cycle peak value, causing most of the values to fit between 0 and 1, except for the false peaks. These two consecutive transformations allowed for a comparison of behaviors among cycles and proved that several epidemic cycles, within the pandemic, have similarities. With these first steps, it is possible to estimate the duration and general behavior of a local episode, even though this, in absolute terms, does not present the same number of deaths or duration as a similar cycle. What remains approximately constant are the proportions of similar cycles. This technique has been applied with great success in the performance prediction of professional athletes and teams [21].

**Figure 10:**
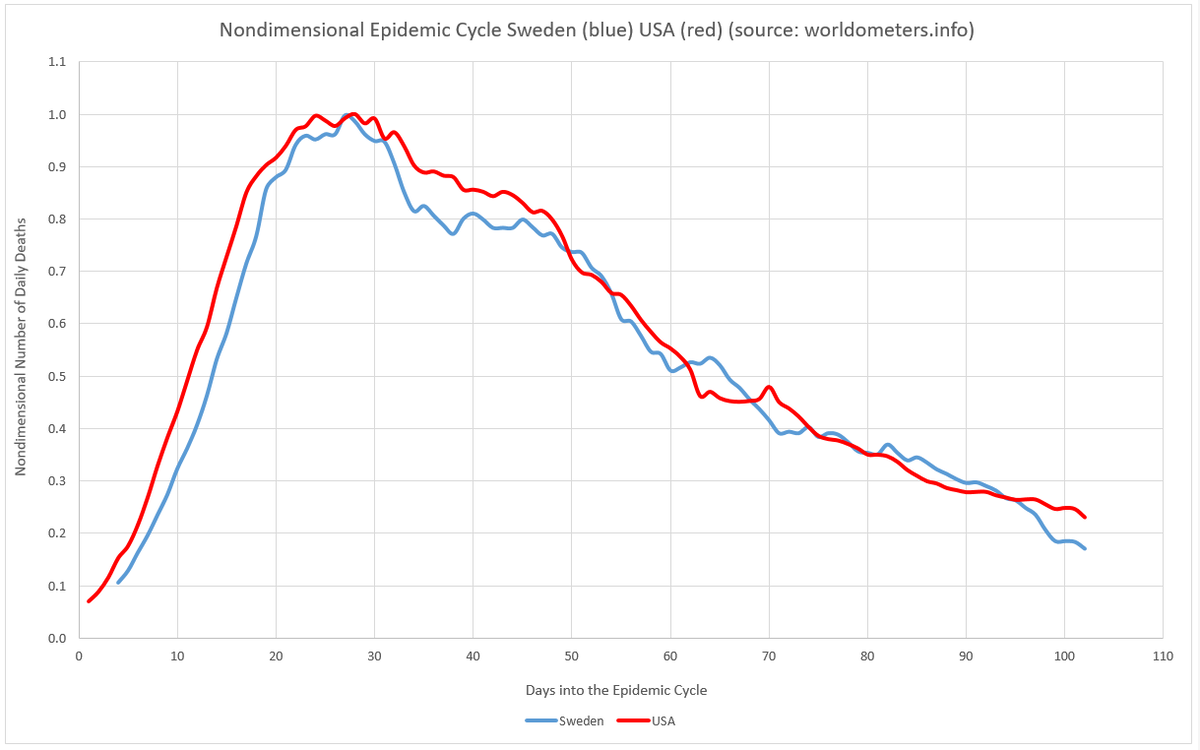
Comparison of epidemic cycles: Sweden and the United States. Source: Worldometer [15].

By the time the analyses were done, the three countries considered in this paper presented more advanced cycles, so no predictions were made for them; instead, their cycles were used to perform analysis on other countries, regions, and cities. For instance, Figure 10 presents the similarity of the United States’ and Sweden’s cycles. A complete set of predictions for Brazil, the state of Rio de Janeiro, and the city of Rio de Janeiro, as well as a measurement of the performance of the model, are presented in Multimedia Appendix 3. In addition, as seen in De Carvalho and De Carvalho [12], it is possible to find many other comparisons and predictions between cities, regions, and countries using this method.

The analyses of the other variables considered in the framework for Germany, Italy, and Sweden are presented in the next sections. The data for this part of the study were also collected from the Johns Hopkins University’s website [17] on the declared dates.

The expressions developed in equations 1 to 5 do not explicitly take into account the incubation period, with the instantaneous rate of change, or daily increase in number of registered infected individuals, calculated as defined in equation 5. For the sake of thoroughness, three simulations were performed, for an incubation period of 5, 10, and 15 days. This was achieved by redefining the expression (1+*b*) for a new set of parameters, basically dividing the total number of reported cases for a given day by the values registered in 5, 10, and 15 days before. In that way, the term (1+*b*) would now reflect the incubation period over R_t_. All simulations yielded zero (0%) change, to the fourth significant figure. Therefore, it is assumed that the described method is inherently insensitive to incubation period variations or influence, reinforcing its simplicity and robustness. The data and calculations are in Multimedia Appendix 4.

### Germany

#### Reproduction Numbers

**Figure 11:**
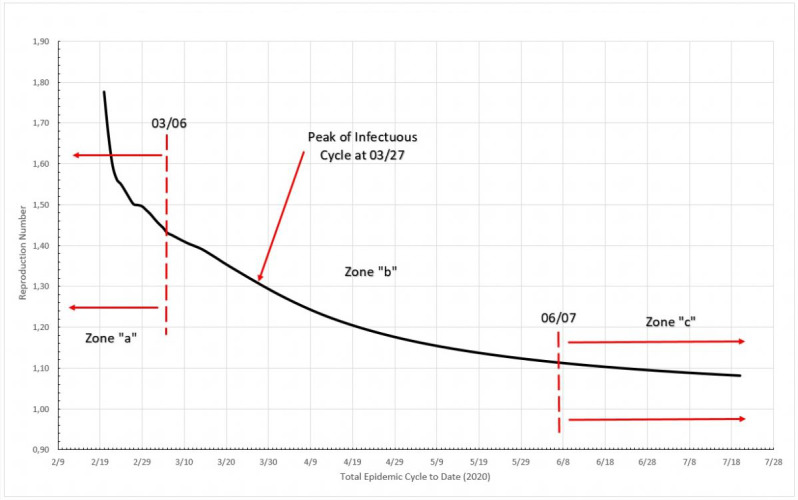
Total epidemic cycle in Germany, using the daily number of infected people. Source: Johns Hopkins University [17].

In Figure 11, three distinct zones are formed. Zone “a” is in the very beginning of the cycle, and the reproduction number varies from 1.10 to 1.48 from one day to the next; this is probably only the reflection of large initial variation in numbers but only if we limit this zone to no more than 5% of the MAMI peak value. It is easy to notice that the figures bear small influence on the overall disease behavior. Zone “b” describes the transmission during the critical disease cycle (from March 6 to June 7), where a rapid increase in daily cases stops only around the peak than drops steadily toward the end. This is the most lethal period of the epidemic cycle, and it is considered over once a 5% peak level is reached again. The remaining time, zone “c,” is the residual cycle that appears in all countries and places facing the COVID-19 crisis. In absolute values, the reproduction number for the critical period starts with a value of 1.30 and drops continuously toward 1.00, although never quite reaching it (at the time this paper was written).

#### Subnotification

**Figure 12:**
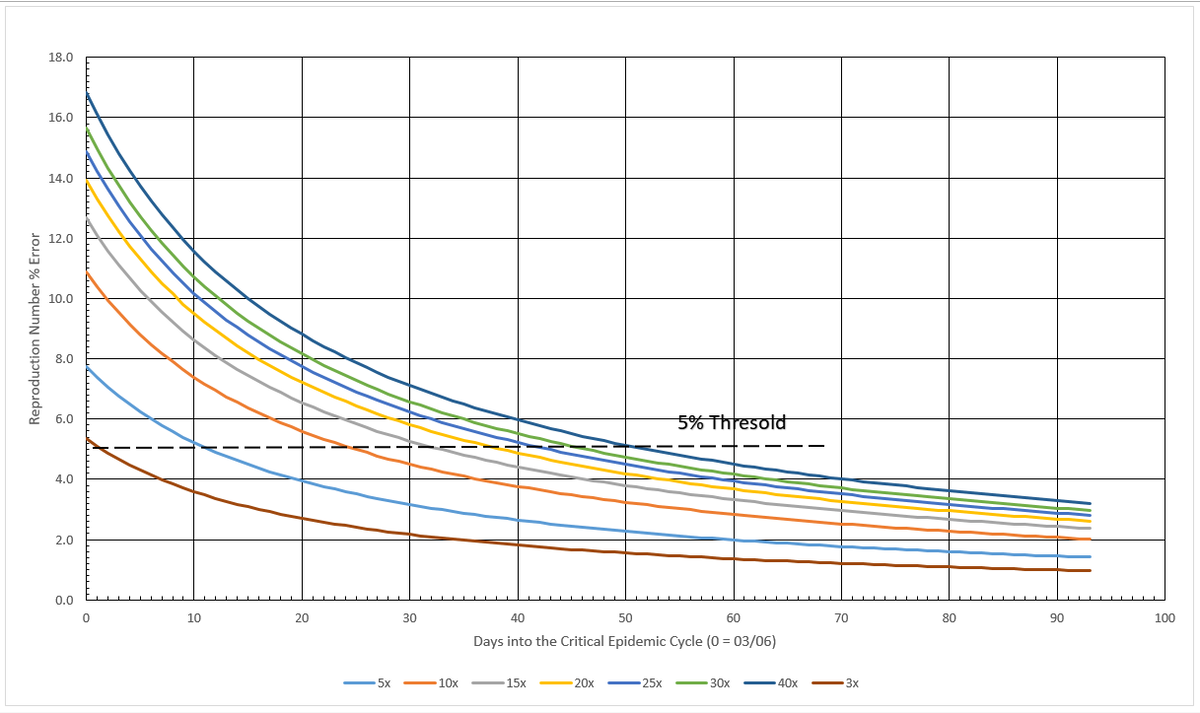
Subnotification effect on reproduction number in Germany during the critical epidemic cycle. Source: Johns Hopkins University [17].

An arbitrary threshold line representing a 5% error was drawn in Figure 12. This limit shows that after the 50th day into the German critical cycle (the one between 5% of the peak value, before and after it), regardless of the amount of subnotification, the error of the calculated reproduction number is no greater than 5%, as presented in Table 3. At the other extreme, a 3x subnotification essentially does not induce errors greater than 5% on the reproduction number, at any time during the critical cycle. A maximum error of 16.84% is estimated for the worst case scenario simulated here, a 40x subnotification, and the first day into the cycle. In overall, subnotification appears to have no significant impact in Germany’s official infected numbers. Subnotification also seems to have more impact in the very beginning of a given cycle but becomes irrelevant toward the end.

**Table 3:** Errors associated with ignoring the existence of subnotification in the epidemic cycle.

Subnotification	Max error (%)	Min error (%)	Days until ≤5%	Error (%) at peak day
3x	5.34	0.97	2	2.64
5x	7.73	1.41	12	3.85
10x	10.87	2.02	25	5.46
15x	12.66	2.37	33	6.39
20x	13.91	2.62	39	7.05
25x	14.87	2.81	43	7.55
30x	15.64	2.97	47	7.96
40x	16.84	3.21	52	8.60

#### Total Number of Infected

**Figure 13:**
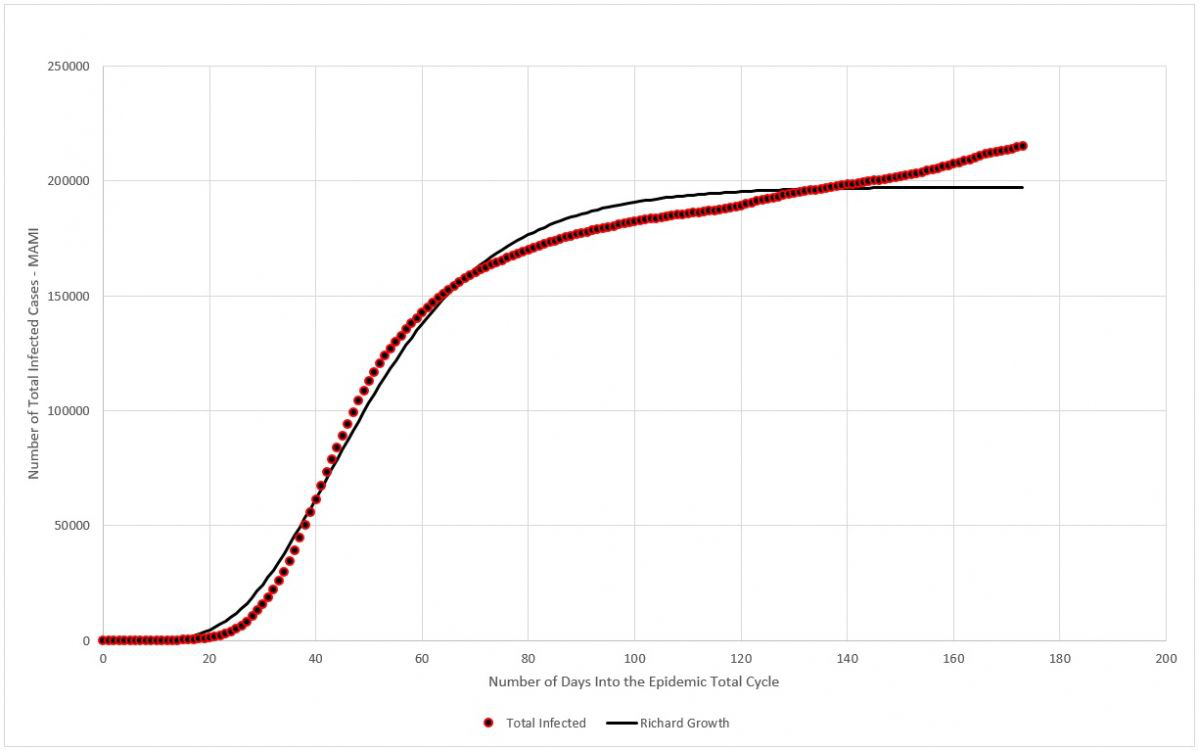
Total number of infected (moving average method–initial value [MAMI]) compared to the Richard growth model prediction for Germany. Source: Johns Hopkins University [17].

Data collected for Germany from February 15 to July 20 were plotted in Figure 13. The blue dots represent the daily registered infected cases submitted to MAMI, and the red continuous line represents the Richard growth model curve, drawn using parameters determined by the MAMI data.

As discussed previously, the German critical epidemic cycle started on March 6. Using curve-fitting data from Table 4, Table 5 shows that the first case must be recorded 89 days before that, with X_3_ indicating that the first case of the total epidemic cycle occurred around December 8, 2019.

**Table 4:** Curve-fitting data.

Parameter	Value
a	197,372.97
b	–5.2260
c	0.0587
d	4.4208×10^-4^

**Table 5:** Epidemic parameters determined using curve-fitting data from Table 4.

Epidemic parameter	Value
X_1_	197,373
X_2_	5.87^a^
X_3_	89
r^2^	0.9958

^a^Percent.

#### Impact of Incubation Period

**Figure 14:**
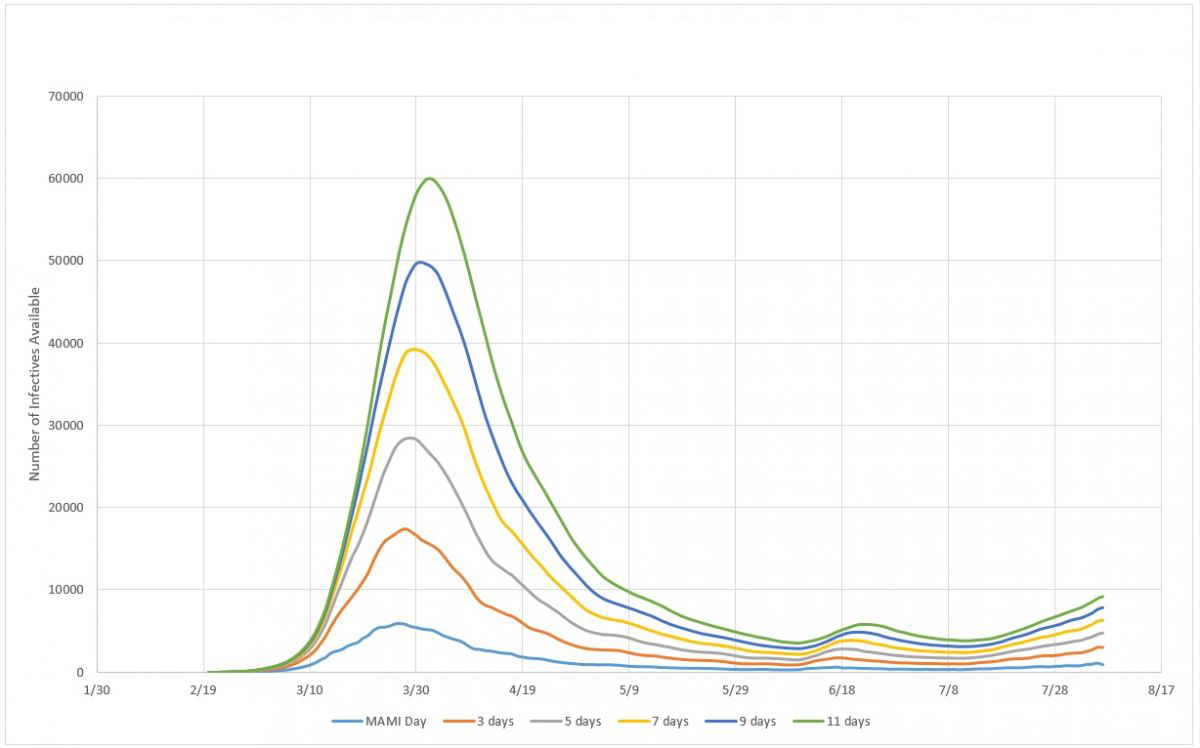
Infected person inventories for 3, 5, 7, 9, and 11 days of incubation, compared to MAMI (moving average method–initial value) for Germany. Source: Johns Hopkins University [17].

In this section, we approach the model of formation of an infected persons inventory for the three countries considered. Simulations were made for incubation cycles of 3, 5, 7, 9, and 11 days. Inventories were calculated according to equation 12 and plotted together with the MAMI of detected cases. Figure 14 presents the subnotification study for Germany.

### Italy

#### Reproduction Numbers

**Figure 15:**
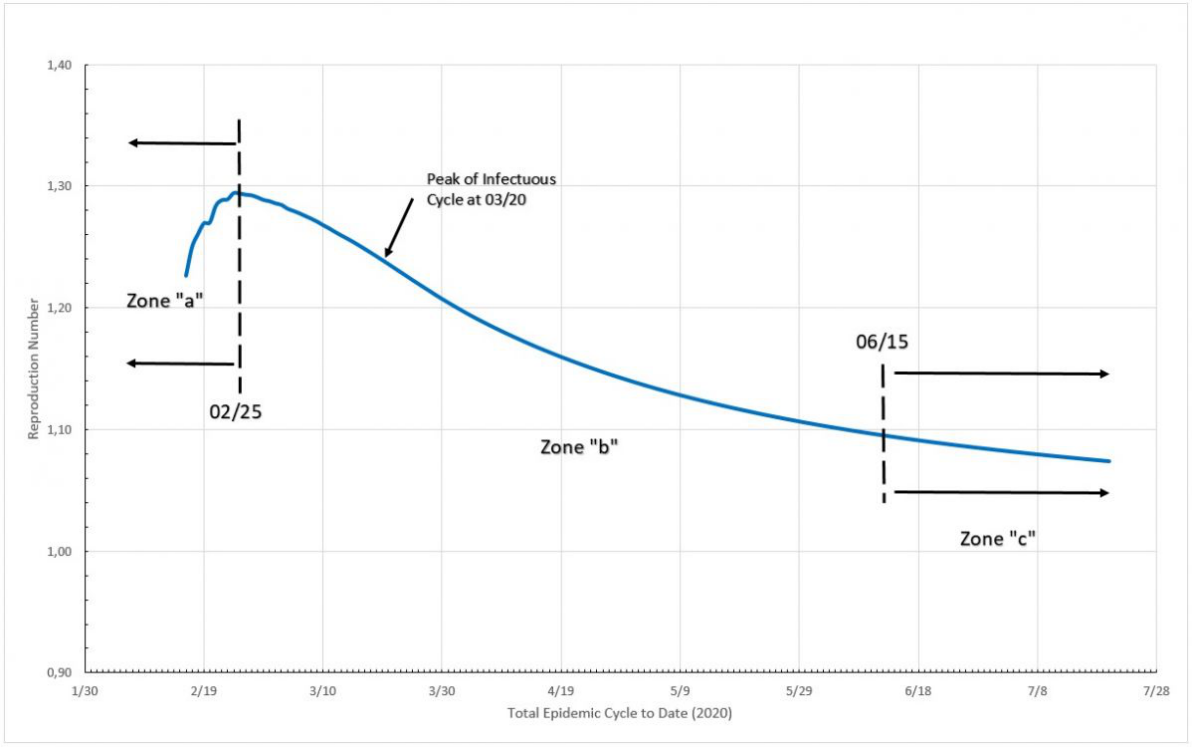
Total epidemic cycle in Italy, using the daily number of infected people. Source: Johns Hopkins University [17].

It can be seem in Figure 15 that three distinct zones are formed. Zone “a” is in the beginning of the cycle, and the reproduction number varies from 1.78 to 1.44 from one day to the next; once again this is probably simply the reflection of large initial variation in number, but this zone is limited to no more than 5% of the MAMI peak value. It is easy to notice that the figures bear small influence in the overall disease behavior. Zone “b” describes the transmission during the critical disease cycle (from February 25 to June 15). This is the most lethal period of the epidemic cycle, and it is considered over once a 5% peak level is reached again. The remaining time, zone “c,” is the residual cycle. In absolute values, the reproduction number for the critical period starts with a value of 1.44 and drops continuously toward 1.12.

#### Subnotification

**Figure 16:**
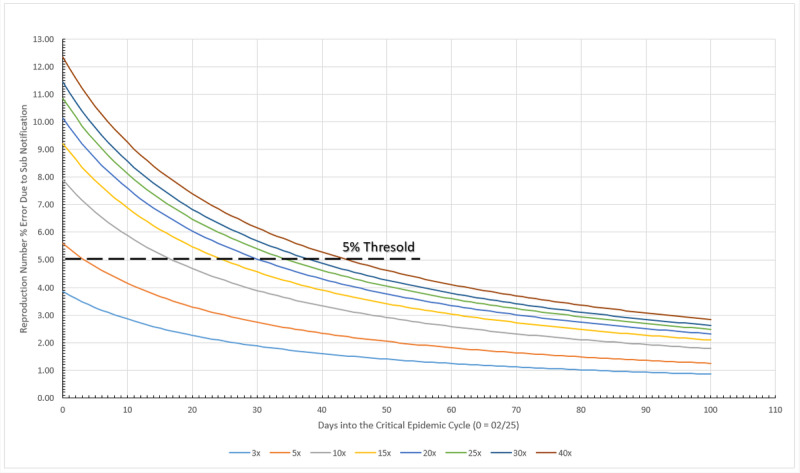
Subnotification effect on reproduction number in Italy during the critical epidemic cycle. Source: Johns Hopkins University [17].

Subnotification in Italy is presented in Figure 16. The 5% limit tells that after the 44th day into the Italian critical cycle, regardless the amount of subnotification, the error of the calculated reproduction number is no greater than 5%, as shown in Table 6. At the other extreme, a 3x subnotification essentially induces no errors larger than 5% on the reproduction number, in any time during the critical cycle, and 5x barely disturbs it. A maximum error of 12.34% is estimated for the worst case scenario simulated here, a 40x subnotification, and the first day into the cycle. Overall, subnotification appears to have no significant impact on Italy’s official infected numbers, as in the previous two cases. Subnotification also has more impact in the very beginning of a given cycle but becomes irrelevant toward the end of it.

**Table 6:** Errors associated with ignoring the existence of subnotification in the epidemic cycle for Italy.

Subnotification	Max error (%)	Min error (%)	Days until ≤5%	Error (%) at peak day
3x	3.85	0.85	N/A^a^	2.09
5x	5.59	1.25	4	3.05
10x	7.89	1.78	17	4.33
15x	9.22	2.09	25	5.07
20x	10.15	2.31	31	5.60
25x	10.86	2.48	35	6.00
30x	11.44	2.62	39	6.33
40x	12.34	2.84	44	6.85

^a^N/A: not applicable.

#### Total Number of Infected

**Figure 17:**
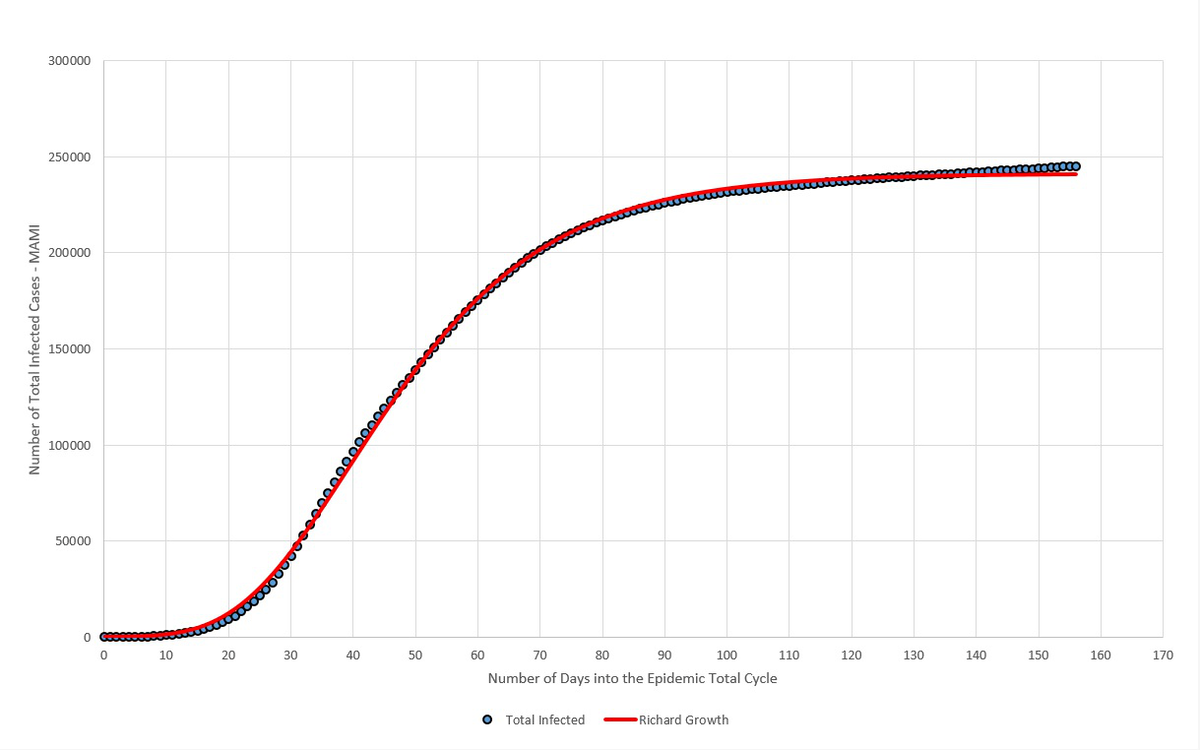
Total number of infected (MAMI [moving average method–initial value]) compared to the Richard growth model prediction for Italy. Source: Johns Hopkins University [17].

Data collected for Italy from February 15 to July 20 were plotted in Figure 17. The blue dots represent the daily registered infected cases submitted to MAMI, and the red continuous line represents the Richard growth model curve, drawn using parameters determined by the MAMI data.

The Italian critical epidemic cycle started on February 25. Using curve-fitting data from Table 7, Table 8 shows that the first case must be recorded 86 days before that, with X_3_ indicating that the first case of the total epidemic cycle occurred around December 1, 2019.

**Table 7:** Curve-fitting data.

Parameter	Value
a	241,148.81
b	–4.8623
c	0.0562
d	8.4600×10^-4^

**Table 8:** Epidemic parameters determined using curve-fitting data from Table 7.

Epidemic parameter	Value
X_1_	241,149
X_2_	5.62^a^
X_3_	86
r^2^	0.9995

^a^Percent.

#### Impact of Incubation Period

**Figure 18:**
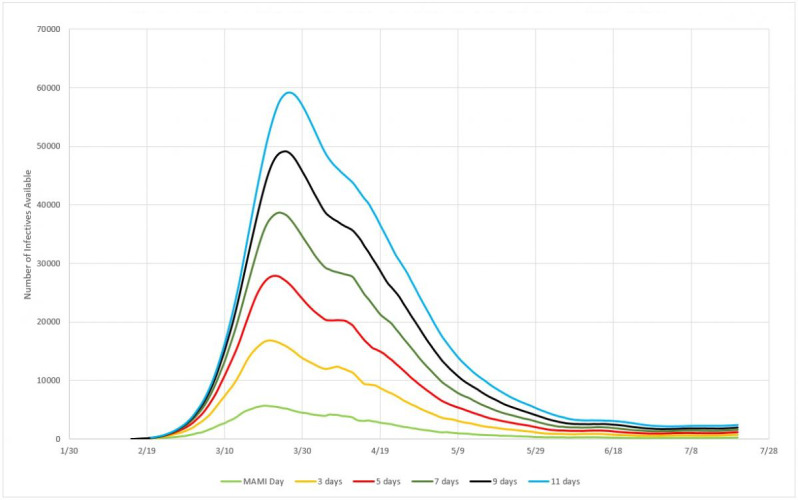
Infected person inventories for 3, 5, 7, 9, and 11 days of incubation, compared to MAMI (moving average method–initial value) for Italy. Source: Johns Hopkins University [17].

Using the same reasoning applied to Germany, Figure 18 presents the inventories of infected persons for Italy.

### Sweden

#### Reproduction Numbers

**Figure 19:**
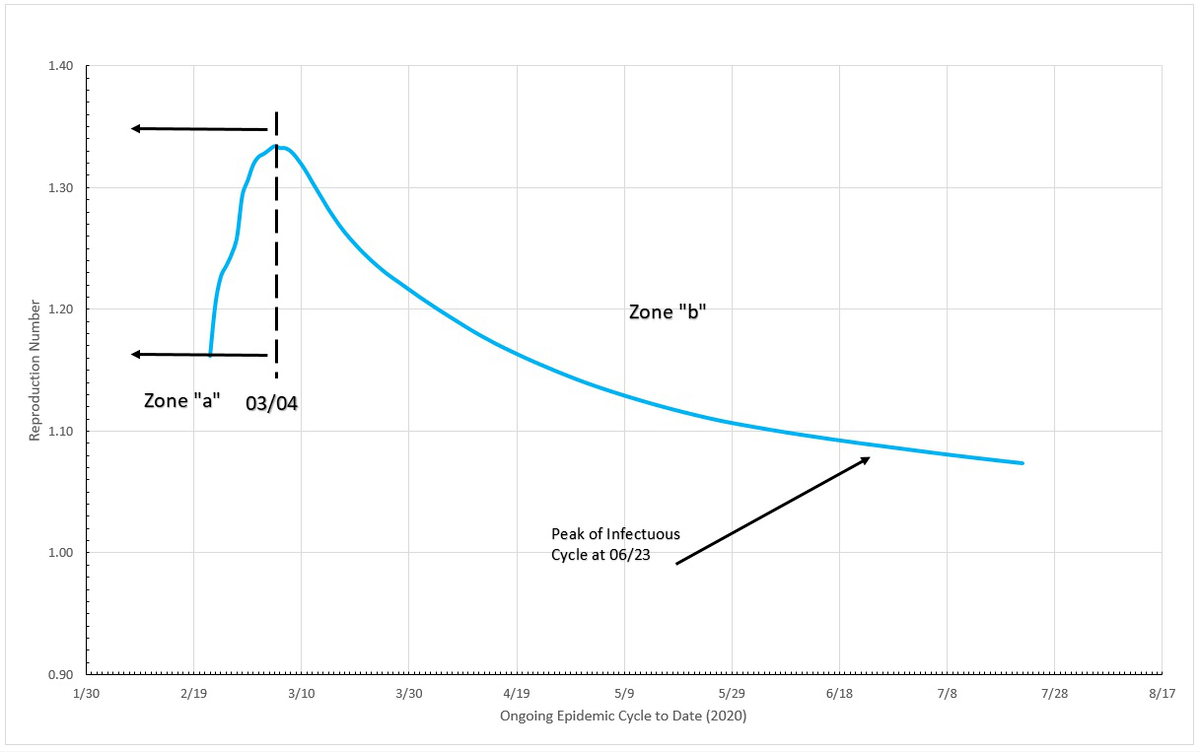
Epidemic cycle in Sweden, using the daily number of infected people. Source: Johns Hopkins University [17].

It can be seen in Figure 19 that two distinct zones are formed, once Sweden is considered, by the 5% criteria an “ongoing” epidemic cycle, although in the present date, close to the end. Zone “a” is in the beginning of the cycle, and the reproduction number varies from circa 1.33 to 1.16 from one day to the next; once again this probably is just the reflection of large initial variation in number, but this zone is limited to no more than 5% of the MAMI peak value. It is easy to notice that the figures bear small influence in the overall disease behavior. Zone “b” describes the transmission during the critical disease cycle (from March 4 onward). This is the most lethal period of the epidemic cycle, and it is considered over once a <5% peak level is reached again. In absolute values, the reproduction number for the critical period starts with a value of 1.16 and drops continuously toward 1.07.

#### Subnotification

**Figure 20:**
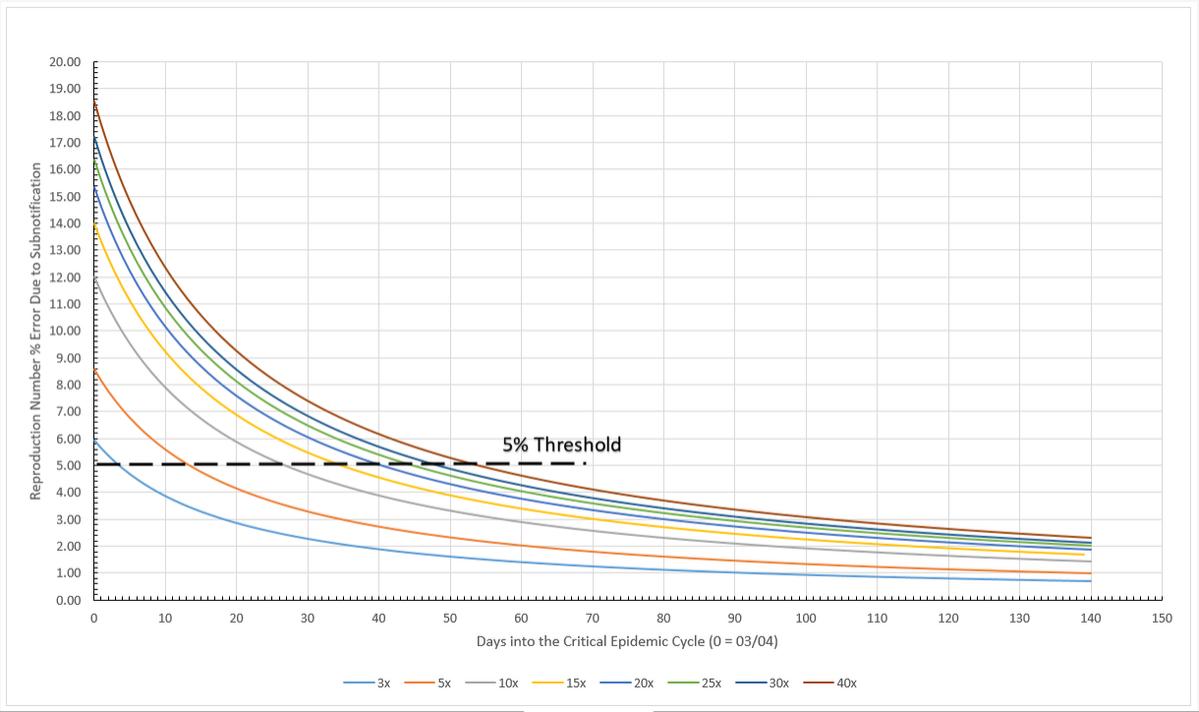
Subnotification effect on reproduction number in Sweden during the critical epidemic cycle. Source: Johns Hopkins University [17].

The subnotification effect in Sweden is presented in Figure 20. The calculated limit tells that after the 54th day into the Swedish critical cycle, regardless the amount of subnotification, the error of the calculated reproduction number is no greater than 5%. On the other extreme, a 3x subnotification essentially induces no errors larger than 5% on the reproduction number, after the fourth day during the critical cycle, as shown in Table 9. A maximum error of 18.53% is estimated for the worst case scenario simulated here, a 40x subnotification, and the first day into the cycle. Overall, subnotification appears to have no significant impact in Sweden. Subnotification also has more impact in the very beginning of a given cycle but becomes irrelevant toward the end of it.

**Table 9:** Errors associated with ignoring the existence of subnotification in the epidemic cycle for Sweden.

Subnotification	Max error (%)	Min error (%)	Days until ≤5%	Error (%) at peak day
3x	5.92	0.69	4	0.85
5x	8.55	1.01	14	1.24
10x	12.01	1.45	27	1.77
15x	13.97	1.70	35	2.08
20x	15.33	1.88	41	2.30
25x	16.37	2.02	45	2.46
30x	17.22	2.13	49	2.60
40x	18.53	2.31	54	2.82

#### Total Number of Infected

**Figure 21:**
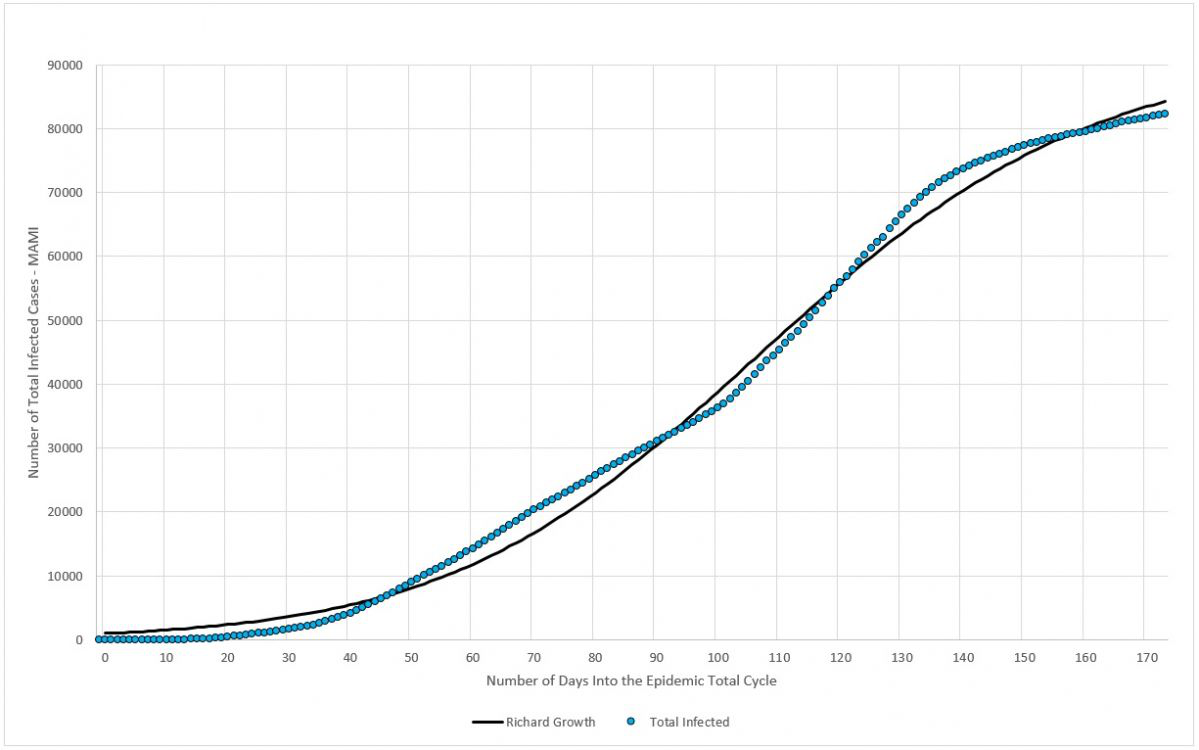
Total number of infected (MAMI [moving average method–initial value]) compared to Richard growth model prediction for Sweden. Source: Johns Hopkins University [17].

Data collected for Sweden from February 15 to July 20 were plotted in Figure 21. The blue dots represent the daily registered infected cases submitted to MAMI, and the red continuous line represents the Richard growth model curve, drawn using parameters determined by the MAMI data.

Previously, it was shown that the Swedish critical epidemic cycle started on March 4. Using curve-fitting data from Table 10, Table 11 shows that the first case must be recorded 98 days before that, with X_3_ indicating that the first case of the total epidemic cycle occurred around November 27, 2019.

**Table 10:** Curve-fitting data.

Parameter	Value
a	92,538.59
b	3.4050
c	0.0348
d	7.5514×10^-1^

**Table 11:** Epidemic parameters determined using curve-fitting data from Table 10.

Epidemic parameter	Value
X_1_	92,539
X_2_	3.48^a^
X_3_	98
r^2^	0.9958

^a^Percent.

#### Impact of Incubation Period

**Figure 22:**
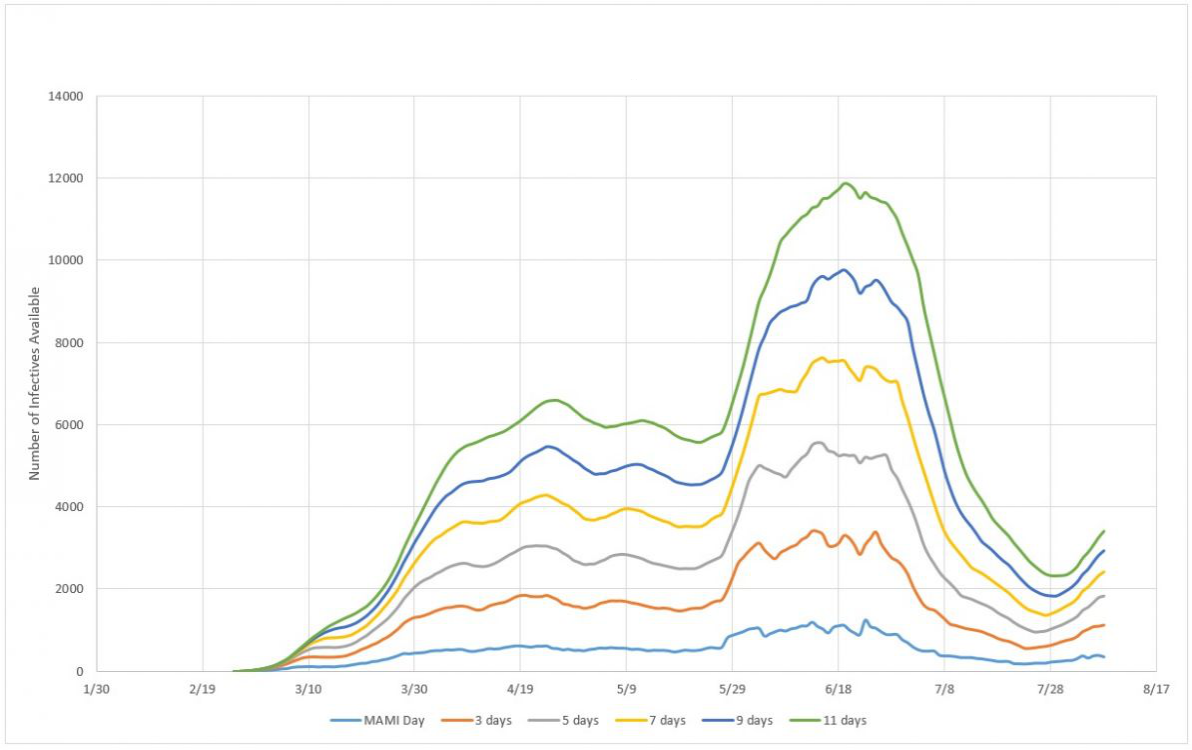
Infected person inventories for 3, 5, 7, 9, and 11 days of incubation, compared to MAMI (moving average method–initial value) for Sweden. Source: Johns Hopkins University [17].

Accordingly, Figure 22 presents the predicted inventories of infected persons for Sweden.

One cannot take the assumptions used to derive equation 12 as deterministic, considering that it describes a perfect “production” system. However, there is no biological system that behaves in such a perfect and deterministic way. Therefore, the data shown in Figures 9, 13, and 17 are not conclusive by themselves, given the imperfections of the contamination paths, or the considered “production system,” should be taken into account. In other words, the efficiency of the transmission system must be evaluated, as done in the Discussion session.

## Discussion

### MLCE Control Performance

**Figure 23:**
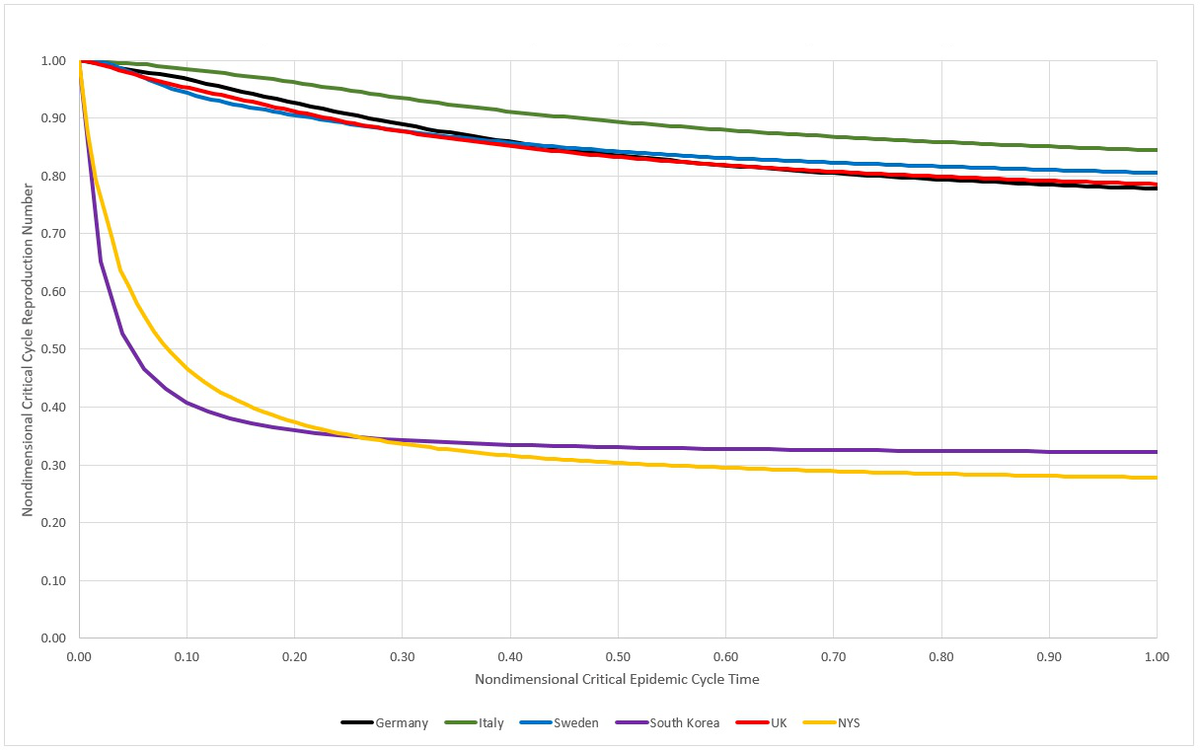
Nondimensional critical epidemic cycle for Germany, Italy, Sweden, South Korea, the United Kingdom, and New York State (NYS). MAMI: moving average method–initial value. Source: Johns Hopkins University [17].

Using the definition of MLCE, a comparison of the three studied countries was performed. As parameters, it were applied an interval within the 5% limits and the nondimensional time calculated by dividing the day numbers by the total MLCE duration, for each country. For the reproduction number, all the values were divided by the largest value found in the MLCE interval. All these transformations allow us to estimate how efficient the disease control measures used in each country were. In order to enrich the comparative analysis, Figure 23 presents the data from the three countries studied here and also from the United Kingdom, South Korea, and the state of New York. Additional details on this and other comparisons can be found in De Carvalho and De Carvalho [13]. Sweden and New York State were considered as still having an open MLCE by the time of the data analysis; therefore, the end of the cycle considered was the day of data collection (July 22, 2020).

Figure 23 shows that Italy was, in relative terms, the most unsuccessful place in reducing reproduction numbers, although not by a large margin. Germany and the United Kingdom exhibited the same performance where the R_t_ fell slowly but steadily. South Korea and New York State achieved a large drop in the early stages of the critical cycle, but after that the R_t_ became more or less constant.

### Efficiency of the Infection System

**Figure 24:**
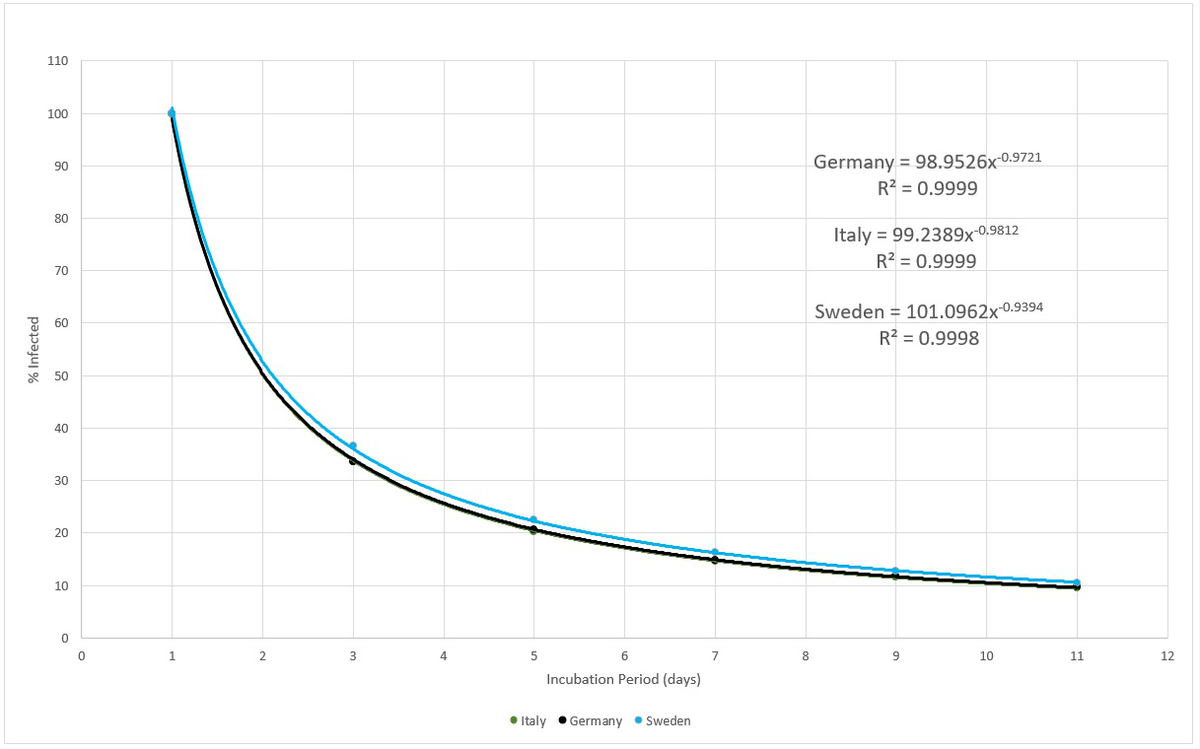
Number of days of incubation versus the percentage of serious and severe COVID-19 infections. Source: Johns Hopkins University [17].

According to the experimental data obtained, the efficiency, or the capacity for spread, of the biological system here described, that is, SARS-CoV-2, has a power function form, as shown in Figure 19. Although the three countries analyzed here present very different epidemic cycles, the percentage of people infected compared to the incubation period varies very little. This probably reflects that the incubation period is in fact a constant value. Figure 24 shows that, for example, for a 5-day incubation period, the percentage of people who were exposed to the virus and displayed symptoms severe enough to prompt them to obtain medical care was around 20%. At the other extreme, if the virus had a 11-day incubation period, the numbers of actual cases registered would have indicated a 10% rate of infection in the general population.

This curve, although restricted to only these three countries, covers nations with quite different NPI policies, population sizes, and land masses. It shows that, according to registered cases, SARS-CoV-2 affected a small segment of these populations and at the same proportions. The subnotification effect does not interfere with this curve behavior significantly, as shown by the calculations.

**Figure 25:**
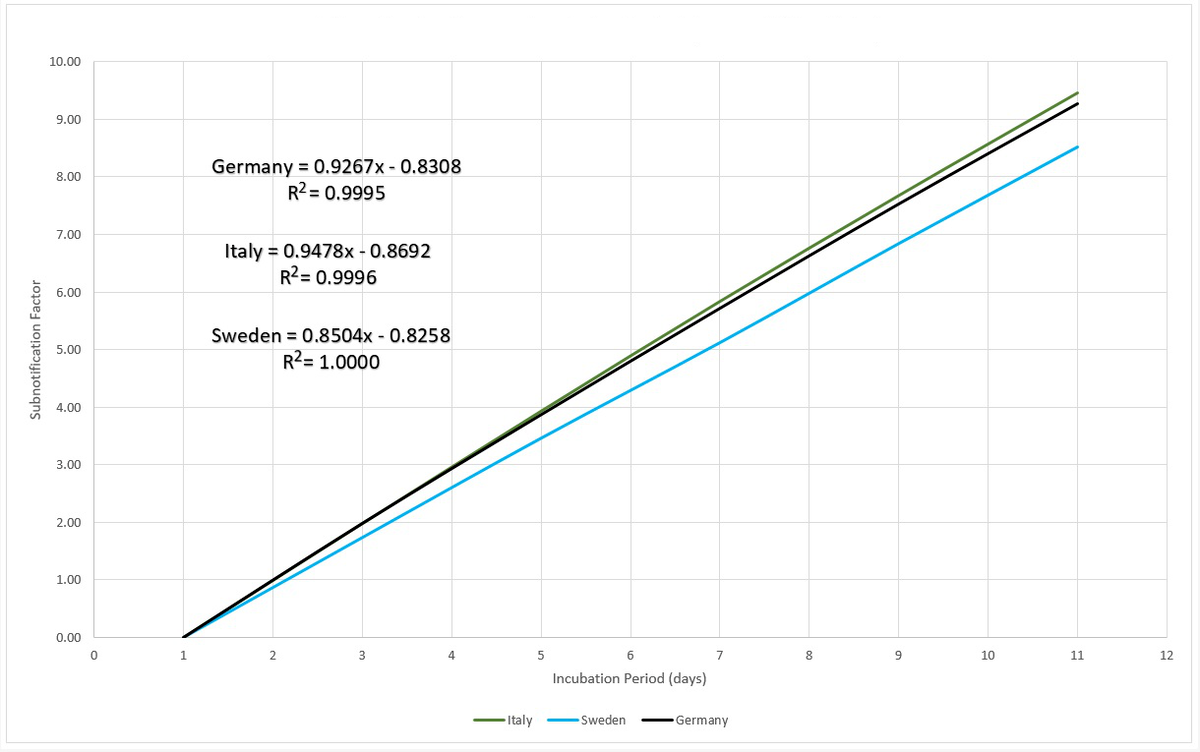
Subnotification factor for the studied countries. Source: Johns Hopkins University [17].

One conclusion is that, putting together equation 12 with the efficiency measurement in Figure 24, the reported subnotification rate of 80% [22], or 20% of people with more serious symptoms, represents 1 in 5 of the infected persons inventory. In other words, there is 5 times more persons in the infective state than detected and reported by the MAMI figures, leading to a 5-day incubation period. The next step is calculating the subnotification estimation, which then becomes straightforward: given the incubation period, how many times should the registered amount be multiplied to correctly express the estimated subnotification? For example, for a 5-day incubation period in Germany, a subnotification around 4 times the registered number of cases in any given day is expected, if 100 were registered as infected and 400 were not. With this rationale, it is possible to compare the subnotification factor with the incubation period for the three studied countries, as presented in Figure 25.

### Other Findings and Conclusions

The early predictions on the progress of the local epidemic cycles of COVID-19 based on Gaussian distribution models and their derivatives, such as the beta distribution, failed to obtain values close to reality, sometimes being very pessimistic, other times being too optimistic. In addition, the nature of the data available for studies requires preliminary numerical treatment, since most of them present the number of daily deaths that occurred on the dates on which they were recorded by the health system and not on those that the deaths actually occurred. Moreover, countries with vast territories and populations should not be treated as a single case, but should be studied regionally, so that the evolution of disease cycles can be clearly understood.

Through the observation of some early cycles, where a peak had already been reached, associated with a consistent reduction in the number of infections, it was possible to identify a triangular shape in these distributions. With the information on the approximate behavior of the variable in question (reproduction number) and the identification of a minimum and maximum, the use of the triangular distribution became clear. After applying this distribution over several local cycles, it was possible to identify similarities between pairs of cycles of localities and regions apparently without direct demographic correlation. Normalization allows you to use an already completed cycle to estimate the behavior of a cycle that is still evolving. The method using the similarity of cycles was able to estimate the end of the cycle up to 34 days before the actual end of the cycle, but requires that there exist a similar cycle. These similarities were confirmed by Kolmogorov-Smirnov tests applied to the data series (Multimedia Appendix 1), demonstrating the hypothesis that the triangular distribution applies to these comparisons and, therefore, is applicable to the prediction of the dimensionless behavior of these cycles. Additionally, understanding the basic behavior of local epidemic cycles allowed for the assessment of the impact of subnotification on calculations.

It is important to note that starting dates influence all the parameters that govern every statistical model used for characterizing the infection. The logistic model together with the model based on the concept of an infected persons inventory can be used to obtain three parameters of the epidemic cycle: the number of total infected, the daily infection rate, and the lag phase, which determines the actual probable onset of the epidemic for the studied countries, thereby solving the problem of noise generation in other parameters by wrongly determined onset dates.

Hence, the experimental framework proposed here offers a set of simple and efficient methods for calculating not only the reproduction number, but also other variables that influence the epidemic cycles and supporting the decision-making process of health authorities, being an interesting tool especially for those places where mass testing is not available. Currently, as the second wave of infections by SARS-CoV-2 emerges, this framework is being applied again in order to definitively demonstrate its efficacy and efficiency.

## Appendix

Multimedia Appendix 1 Data and calculations for the studied local cycles.

Multimedia Appendix 2 Data and calculations for Rt and subnotification.

Multimedia Appendix 3 Extra case study: Brazil.

Multimedia Appendix 4 Data and calculations for the logistic model and the inventory model.

## Abbreviations

MAM: moving average method
MAMI: moving average method–initial value
MLCE: most lethal cycle of the epidemic
NPI: nonpharmaceutical intervention
R_0_: reproduction number
R_t_: effective reproduction number
