# Supplementary material for: A Framework for a Statistical Characterization of Epidemic Cycles: COVID-19 Case Study
Source: JMIRx Med. 2021 Mar 18;2(1):e22617. doi: 10.2196/22617 (PMC8078446; doi:10.2196/22617)
Supplement: Multimedia Appendix 3 [file xmed_v2i1e22617_app3.docx]

**APPENDIX A**

The lessons learned in the analysis of patterns between countries and cities in the Northern Hemisphere were used to identify and analyze patterns in Brazil, whose epidemic cycle started later.

**Brazil, State of Rio de Janeiro and the City of Rio de Janeiro**

Brazil, if seen as a whole, has the same slow cycle of rise as India and Mexico; and a long cycle duration like the USA. These are all countries with large territorial extensions and population concentrations. Russia, Australia and Canada are large countries, but with sparse populations, therefore with another type of epidemic cycles. We understand that countries like Brazil should be seen and dealt in terms of metropolis or regions, with regard to the study of epidemic cycles, since regions that started their cycles at the beginning of the pandemic, are already at the end or close to it, and those who have only had residual cases so far can start the acute cycle at any time. Seen as a whole they seem to be a long cycle and different from all those presented here in this work, but if seen individually, they are quite similar to the “triangular” cycles presented previously. Countries with a large population and continental dimensions, may have their epidemic cycle curve built from the convolution of the curves of the cities with the largest population.

The data sources used in this study for Brazil are the Ministry of Health's website (<https://covid.saude.gov.br/>) and National Council for Justice’s Transparency Portal, or TP for short (<https://transparencia.registrocivil.org.br/especial-covid>). The Ministry of Health (MoH) publishes data on deaths on the registration dates, in the same way as several other countries presented here, implying the appearance of the inevitable “saw” aspect in the graphics. The second site presents the number of deaths on the dates when they actually occurred, but inevitably suffer from a delay in updating values, due to the time required for families to officially register the deaths and this information become part of the system. The numerical tools presented minimize the effects described and allow a clearer view of the epidemic in Brazil.

Ministry of Health

The Ministry of Health publishes in a daily basis the sum of the number of deaths received and verified, from the state health departments and the Federal District. These are the numbers recorded that day. The death may have happened many days before, but it will be accounted on the date of registration. The effect of this is sharp drops on weekends and extreme peaks in the middle of the weeks. However, these numbers reflect the average (MAMI) of the true values of deaths in the country. If there are adulterations of values, this occurs in the origins of the values and not in these totals, therefore this work will consider this source as a valid one. Confirming the validity of this source, there is the following reference, whose values are very similar to those of the MoH and tend to converge over time, implying not only the veracity of the figures presented, as well as the absence of significant underreporting in the MoH numbers.

Transparency Portal

Presented as an initiative by the Brazilian Civil Registry Offices, this website compiles death registration data across the country, with special attention to COVID-19 data. The data is updated about three times a day. It is delayed in relation to the real value, due to the obvious need of families to register deaths on dates after the actual events. Its values ​​for cities such as Rio de Janeiro vary markedly only in the most recent days (up to four days on average), however considering Brazil as a whole, and its more distant regions, this variation occurs for a longer time. Thus, there are two variations to be observed in this database, the first being how long it takes a given day on the calendar to reach a given value that will be considered to have “converged”, that is: from a given date it will only will change residually, not affecting any study done, the same can be seen in Worldmeter’s portal. The second variation stems from the fact that the calendar continues normally and the days advance, with new deaths being launched and the most recent days are, by definition, underreported. This work evaluated how long it takes for a given value to reach stability, which was defined as being when it stopped varying more than 1% within a week. Appendix A presents a comparative study between the two portals.

Time to Value Convergence - City of Rio de Janeiro

In order to assess the time it takes a certain death curve to reach stability, with stability defined here as that level that varies less than 10%, the values ​​of the city of Rio de Janeiro were monitored. Evaluating the graph in Figure A1, the first observation is that only the last four days vary by more than 10% over the course of a week. In other words: after two weeks there is no more significant change in the values ​​presented, except for the last four days. From the first day (blue, 23 of June) to the second day observed (29 of June), six days pass and although on average the values ​​are less than 10% (6.0, calculated), there are isolated cases that varied more than what is this. However, between 06/29 and 07/07, six days pass again and now the average variation is 0.3%, if the last four days are excluded. Thus, it can be said that for the city of Rio de Janeiro, currently, it is enough to eliminate the last four days to make an accurate projection of the evolution of the epidemic cycle in the city. In the same way, you can study and evaluate any and all cases covered by the Transparency website.


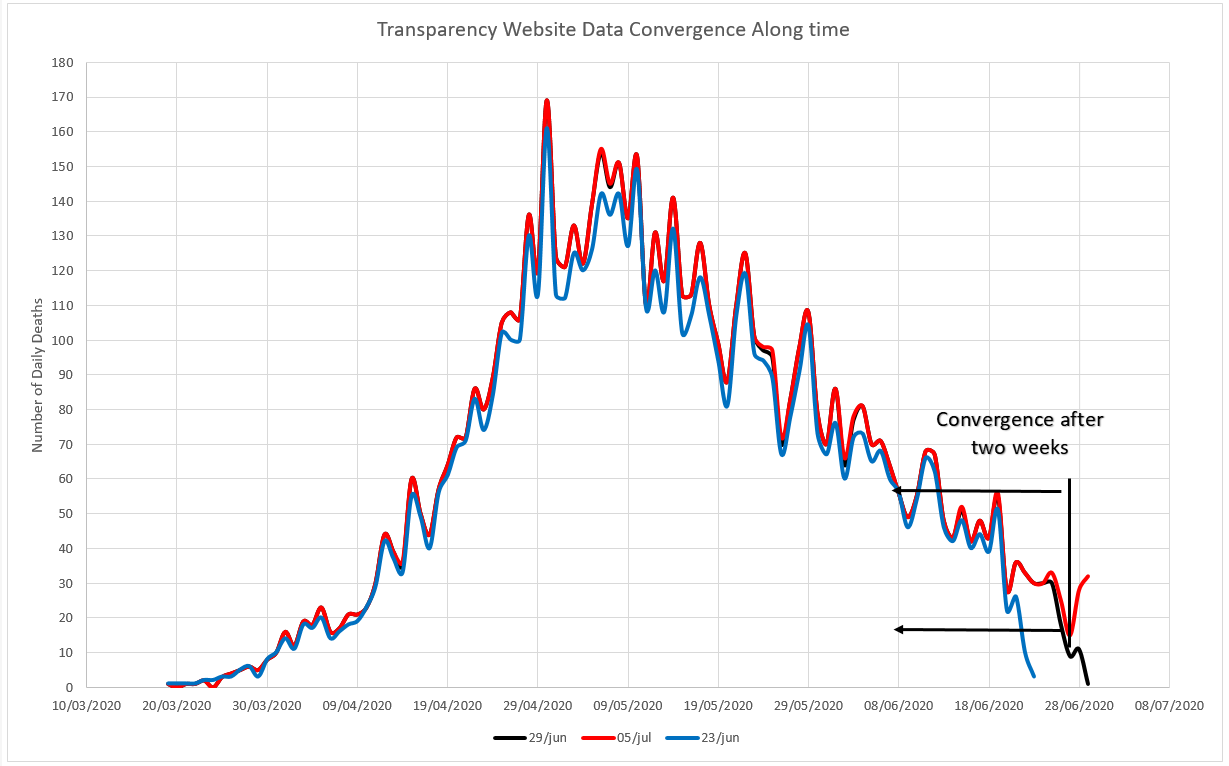


Figure A1 – Daily death figures data for the City of Rio de Janeiro, in three days.

Time to Value Convergence - Brazil

This work considered that variations in the number of deaths in a given day that are less than 5% are negligible and that are up to 10% can be accepted without elimination. This implies that the data to be deleted will be that of the last ten days. This can be seen in Figure A2. Comparing the data from the 5th of July (black line) to the 29th of June (red line) it is noted that before that, from the 17th day backwards, the values are below 3% and in average 1%, all with no practical effect on any projection you want to make (Zone A). Even when observing the values of June 24 (blue curve), although the variation occurs above 10% in a period over ten days (Zone B), the shape and proportions of the curve are identical to the two curves on dates later, this indicates that the distribution of later notifications does not change the shape of the curves and that if they are subjected to the numerical methods mentioned above, they can in fact serve as predictions with acceptable accuracy.


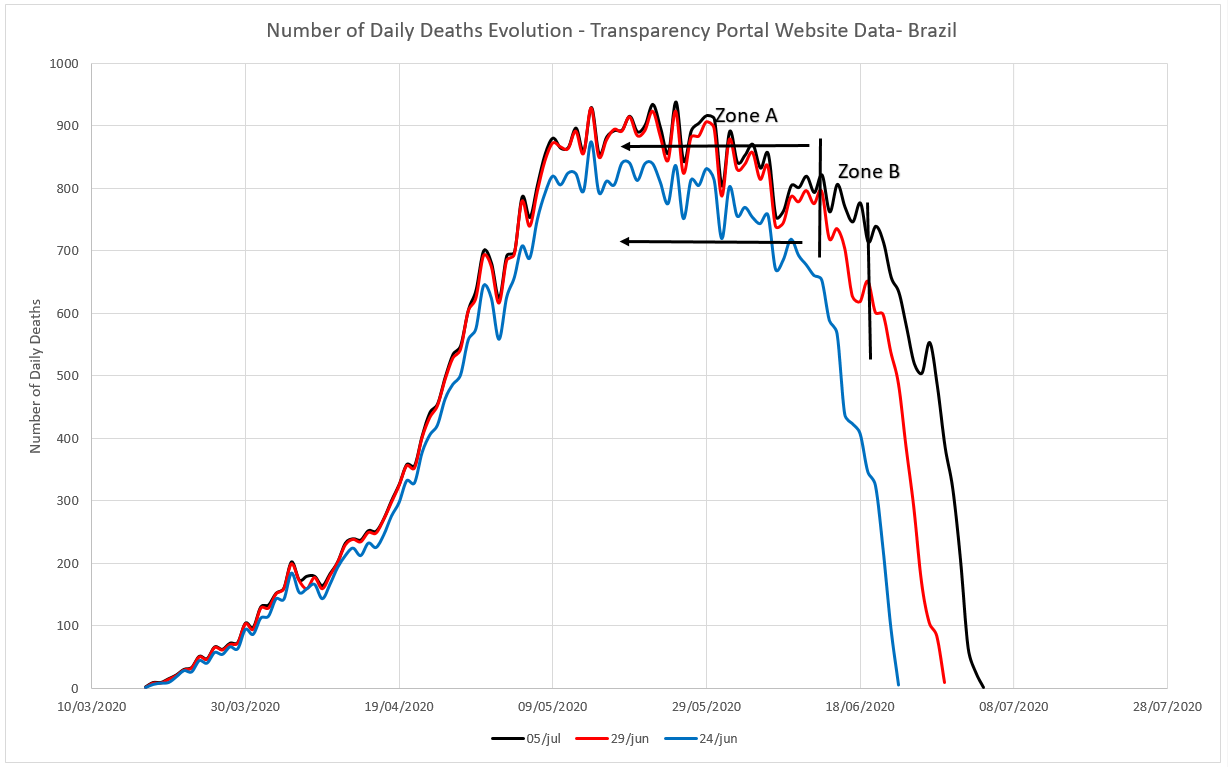


Figure A2 - Daily death figures data for Brazil, in three days.

**Total Deaths**

Brazil

The inevitable delay between the occurrence of physical death and the registry at the registry office has an effect on the count of total deaths in Brazil and in each location. At the end of the epidemic the two values (MoH and Transparency Portal will converge, but during the outbreak, there will always be a delay between the two). The following example was carried out on the afternoon of July 5, 2020. The total number of deaths indicated by the Transparency Portal website was 58155, while the MoH website had the number 64265 deaths. A difference of 6110 deaths (9.5% of the total) that occurred, but have not yet been registered.

City of Rio de Janeiro

In the case of the City of Rio de Janeiro, for data collected on the morning of July 6, 2020, on the two websites mentioned, it reveals that the Ministry of Health points to a total of 6898 deaths in the city so far and the Transparency Portal (TP) indicates 6855, a difference of 43 deaths, that is, about 0.6%, indicating an excellent coherence between the two total values, and the distribution, after the application of MAMI, indicates the existence of the convertibility of the MoH values to the TP, although not as complete as in the case of Sweden, previously described.

**City of Rio de Janeiro Cycle Prediction**

Using the algorithm described in the framework, a forecast of the cycle of the city of Rio de Janeiro was carried out, having the case of Italy as a reference and by extrapolating from the data series, with the elimination of the last values.

City of Rio de Janeiro and Italy

Since Italy was the case chosen to serve as a forecasting model, the predicted values were updated based on the daily values obtained on the TP website, that is: values presented here were collected on the mentioned dates. Although the cycle of ascent in Italy was not similar to that of the city of Rio de Janeiro, the descent cycle was considered to be good enough in the initial moments and this model was adopted, which proved to be correct. It was considered in item 4 that the Italian critical cycle ended when the level of daily deaths decreased until reaching 10.6% of the peak already submitted to MAMI, from then on the residual cycle started. For the city of Rio de Janeiro, after the MAMI, the peak value becomes 143, so 10.6% of this would be 15 deaths. So when the city of Rio de Janeiro reaches approximately 15 daily deaths, the critical cycle can be considered closed. Note that on the forecast day, July 5, the curves met.

In the first assessment, made on June 1, 2020 (dark blue curve, Figure A3), the concordance between the proportions graph of Italy (black curve) and the city of Rio de Janeiro coincides until May 21, after which there is a great divergence, but it is possible to note that: a) the cycle is still far from the end and b) the proposed cycle describes the critical episode in the decent as well, for about 1/3 of the period. If the appraiser accepts this period as being sufficient to characterize a valid similarity, it is sufficient to consult the final value of the Italian episode (10.6%) and estimate the date when the Rio de Janeiro’s cycle will reach that value of its peak, as described above.

On June 10 (purple curve) as well as on all dates used in this graph for the city of Rio de Janeiro, the last 4 days were marked, which are contained within the ellipses. Observing the data of these 4 final dates of each series guarantees that they will grow and, therefore, become even more similar to the Italian model, as is evident when analyzing the later dates, but at the time of collection they were still at their minimum. The agreement on this curve runs until June 2, then diverges, but more than half of the descent cycle agrees with the Italian, so the forecast becomes quite assertive.

For the data collected on June 17 (green curve), the curve agreement runs until June 7, then diverges slightly until June 10, but the agreement with the Italian is quite obvious and the date foreseen for this cycle, 05 July, is increasingly confirmed. For June 23 (yellow curve), the data coincides until June 16 and the whole curve agrees on more than 4/5 of its extension, confirming the initial estimate of July 5. On 06/29 (blue curve), the data are intertwined with the Italian cycle until June 19, always confirming initial estimates. On 07/05 (red curve) there is a strong interweaving of this data, confirming the end of the cycle by this estimate. The actual data obtained confirms the estimation.

The critical part of the fall cycle in the city of Rio de Janeiro was then estimated to have started on May 4, 2020 and ended on July 5, where it would have reached the value where the residual cycle begins. Thus, the fall cycle lasted 62 days. Joining the 42 climb, it is 104 days in total, that is, a cycle of almost 15 weeks.


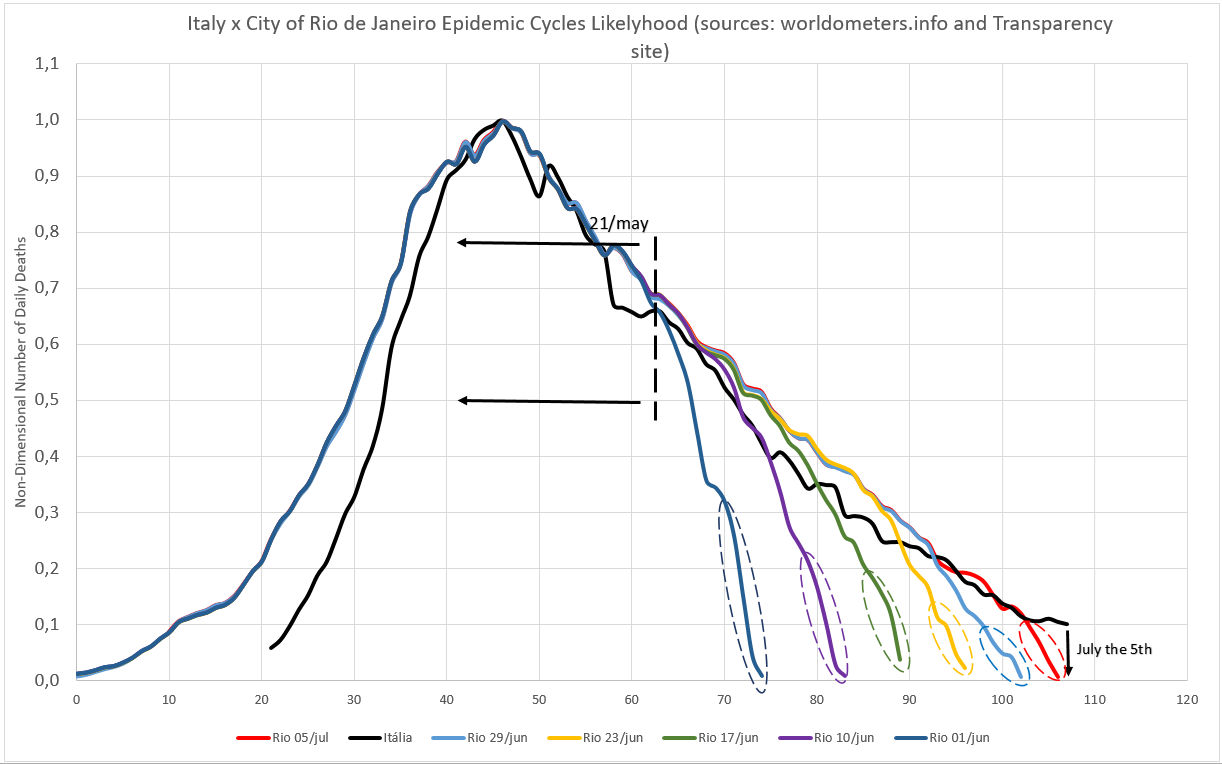


Figure A3 – Several evaluations for the end of the cycle comparing the city of Rio de Janeiro and Italy.

Table A1 – Comparative values determined by Italy and City of Rio de Janeiro epidemic cycles.

| Data Collection Day | Limit of Good Agreement With Italy | Total Days of Good Agreement | Good Agreement and Total Descent Cycle Ratio | Days to the End of the Predicted Epidemic Cycle |
| --- | --- | --- | --- | --- |
| 01/jun | 21/may/2020 | 17 | 0,27 | 34 |
| 10/jun | 02/jun/2020 | 29 | 0,47 | 25 |
| 17/jun | 07/jun/2020 | 44 | 0,71 | 18 |
| 23/jun | 16/jun/2020 | 50 | 0,81 | 12 |
| 29/jun | 19jun/2020 | 56 | 0,90 | 6 |
| 05/jul | 30/jun/2020 | 57 | 0,92 | 5 |

The values in Table A1 indicate that it was already possible to make an accurate estimate for the end of the critical epidemic cycle in the city of Rio de Janeiro on June 1 (35 days before the end), although this contained a greater degree of uncertainty, since only 29% of the descent curve agreed. However, as of June 10 (25 days in advance), with 47% agreement between the curves, the date could already be estimated. As of June 17, there is a very strong agreement between the curves, above 70% and the forecast becomes even safer. If the last 4 days of the series are discarded, as already discussed, it can be noted that the series of July 5 becomes identical to that of Italy from June 30.

Data Extrapolation

To serve as an example, three dates and data that were collected on those dates on the TP website were selected. The data collection dates were 06/23, 06/29 and 07/05/2020. After this, a selection of the peak is made as the last point at which the series rose and from then on it started to decrease, even if in an irregular way. The points (including this peak) are selected excluding the last 4, for the reasons already discussed, submit them to MAMI and apply the linear regression process, where a line is forced to pass through these points. The equation for this line is then equaled to the null value and then the number of days of the epidemic cycle is found, with Day Zero being March 19, 2020, the date of the first death that occurred, which corresponds to a date in the spreadsheet. The values ​​obtained are listed in Table A2. Figure A4 presents the example of the case for the data collected on July 5, 2020. All other cases presented here were calculated in the same way, as already described. It can be seen from the data in Table 5 that the time (in days) between the day the data was observed and the predicted date, is reduced as the end of the cycle approaches. This is achieved when the difference between the predicted day and the day of the data used is zero.


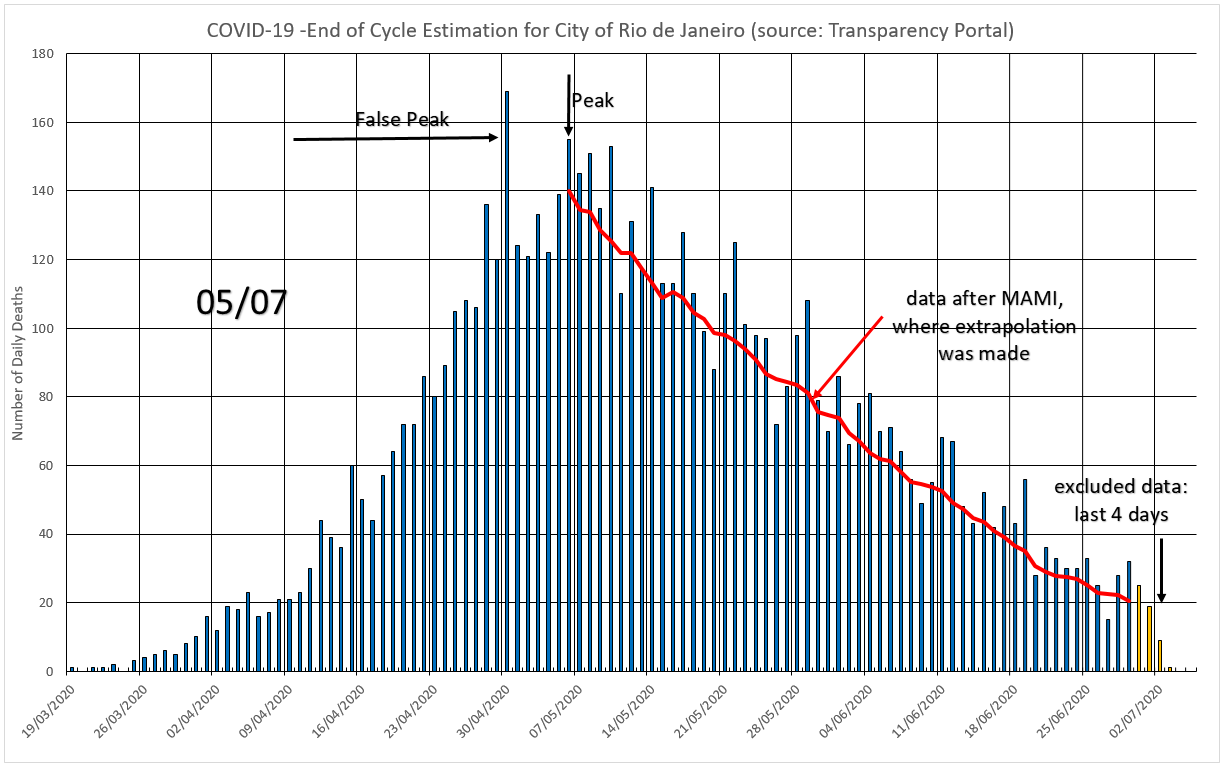


Figure A4 – Data extrapolation for end of cycle estimation using data collected in July the 5^th^, 2020.

Table A2 – Estimation results for several different days.

| Day | Data Fitting Eqn. | R^2^ | Days into the cycle | Estimated Day | Days to the end of the cycle |
| --- | --- | --- | --- | --- | --- |
| 23/06 | y =-2,2263x + 289,68 | 0,9939 | 104 | 01/07/2020 | 8 |
| 29/06 | y =-2,3338x + 304,76 | 0,9961 | 105 | 02/07/2020 | 3 |
| 05/07 | y =-2,2086x + 237,70 | 0,9951 | 107 | 05/07/2020 | 1 |
| 07/07 | y = -2,1864x + 236,24 | 0,9933 | 108 | 06/07/2020 | 0 |

Evaluation of the Rio de Janeiro’s Cycle Duration by Comparison

Previously in this work, several cases whose critical cycles can, at the moment of the analysis, be considered as closed. They are used for an assessment, considering that there is a proportion between the time elapsed between the rise the fall of deaths. Table A3 and A4 present these estimates based on the cases presented previously. Figure A5 shows the Rio de Janeiro’s cycle, which presented twice the time of ascent as the world average (21 against 42 days). On the other hand, the decline was relatively rapid, compared to the rest of the world, although this is due to the long rise. The number of actual deaths (6918) registered at the registry office on 07/07/2020 is within the limits of Table 2, although very close to the lower limit. As for the estimated time, following the proportions listed in Table 1, the values are absurd, since the Rio de Janeiro’s cycle, for all purposes, is at the end.

Table A3 – Cycle duration estimation for the City of Rio de Janeiro, based on Table A2’s values.

| Reference Place | Multiplication Factor | Days up to the peak in Rio | Days up to the end in Rio | Predicted Day |
| --- | --- | --- | --- | --- |
| Spain | 2,1 | 42 | 88 | 02/aug/2020 |
| Germany | 3,2 |  | 134 | 17/sep/2020 |

Table A4 – Number of deaths estimation for the City of Rio de Janeiro, based on Table A3’s values.

| Reference Place | Multiplication Factor | Deaths up to the peak | Deaths up to the end | Predicted Total Deaths |
| --- | --- | --- | --- | --- |
| Sweden | 1,6 | 2588 | 4141 | 6729 |
| Sweden | 3,3 |  | 8540 | 11128 |


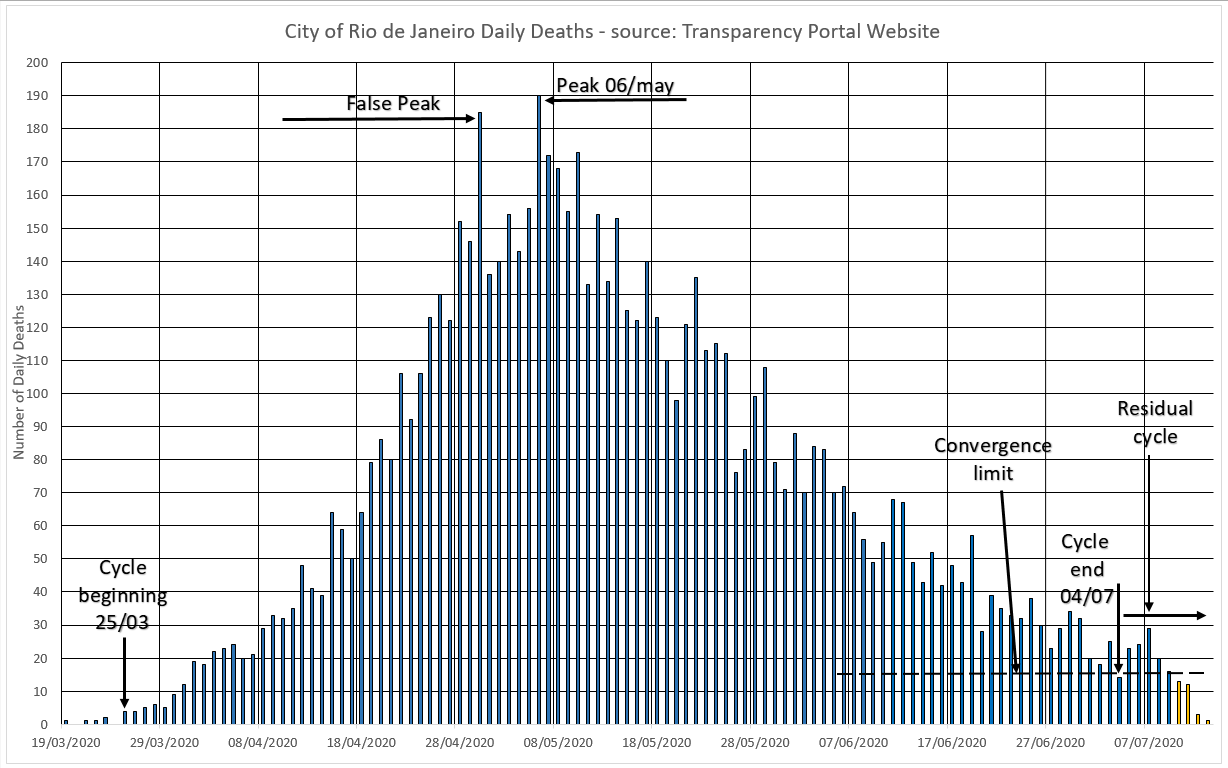


Figure A5 – The Rio de Janeiro’s cycle, “closed” by the time the analysis was done.

**Data Likelihood Verification**

The method mainly claims that is able to predict a given cycle if it is similar to a previously finished one. To verify the mathematical likelihood of the presented cases, the Kolmogorov-Smirnov Test is performed in all pairs of mentioned cycles. Table A5 presents the analysis. Our hypothesis is H_0_ = Sample follows the triangular distribution, with H_1_ = Sample does not follow the triangular distribution.

Table A5 - Kolmogorov-Smirnov Test performed in all pairs of mentioned cycles

| Pair | D | K-S | p-value of test | p>0.05 (α) |
| --- | --- | --- | --- | --- |
| Sweden  USA | 0.133 | 0.941 | 0.338 | Accept H_0_ |
| Spain  NYC | 0.164 | 1.052 | 0.219 | Accept H_0_ |
| Italy  Rio June 1^st^ | 0.150 | 0.954 | 0.322 | Accept H_0_ |
| Italy  Rio June 10^th^ | 0.133 | 0.867 | 0.440 | Accept H_0_ |
| Italy  Rio June 17^th^ | 0.110 | 0.734 | 0.654 | Accept H_0_ |
| Italy  Rio June 23^rd^ | 0.121 | 0.822 | 0.509 | Accept H_0_ |
| Italy  Rio June 29^th^ | 0.132 | 0.909 | 0.380 | Accept H_0_ |
| Italy  Rio July 31^st^ | 0.117 | 0.812 | 0.525 | Accept H_0_ |

**Comparison of Data from the Transparency Portal and the Ministry of Health**

Both databases offer advantages and disadvantages, but both are reliable and over time will converge to similar values. To prove the veracity of this statement, the following procedure was done:

a) The daily data on deaths, released by the MoH were converted into new values ​​by the Moving Average Method with Initial value (MAMI), that is, the average of the values ​​of six days ahead, plus that of the day itself, assumes the first value in the spreadsheet and so on.

b) A curve was then drawn connecting all these points (black curve).

c) The daily data on deaths collected on the Transparency Portal website were listed and a curve linking all these points was drawn (red curve).

d) The two curves were superimposed, for comparison purposes, since they originally occur on different dates (as expected).

e) It is clear that there is a period of agreement between the two curves, which is the beginning of the epidemic, whether in Brazil or in the locality of interest. When moving to the right, the difference increases, consistent with the fact that recent records have not yet been made at the registry offices, but have already occurred at the health departments of municipalities and states, so these deaths “occurred” in the MoH but not in the notaries.


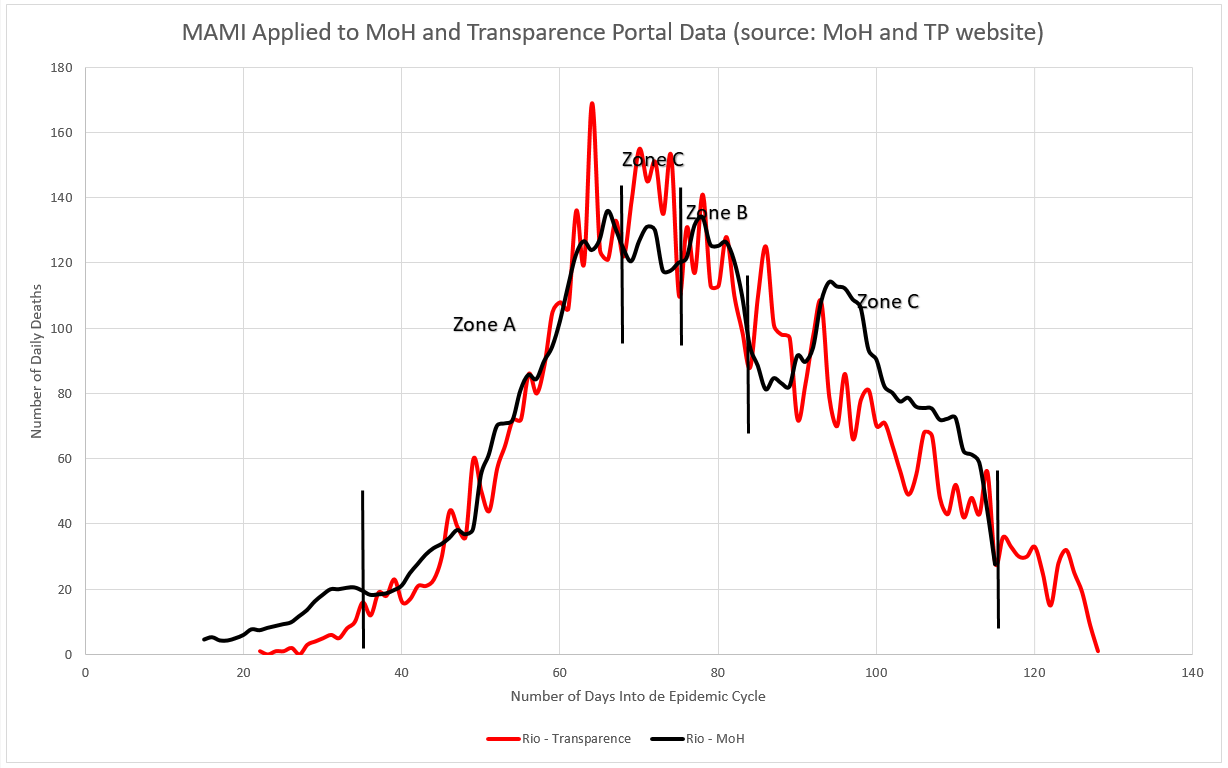


Figure A6 – MoH (black) x TP website (red) data comparison after MAMI.

In Figure A6, Zone A can be seen, where the values of the two sources differ by an average of 7%, although they may present some value outside this range, they are negligible differences. In Zone B, this figure grows to around 9%. In Zone C, the value rises to around 15% which, although not negligible, it is still tolerable in any numerical modeling of human events. There is no equivalence between the last days, which is expected, since the two bases report past values, but with different delays. In order to perform any extrapolation using the MoH values, care must be taken to avoid using data from this final time non-concordance range.

The Brazilian Case

In Figure A7, Zone A can be seen, where the values of the two sources differ by an average of 1%, although they may present some value outside the unitary range, they are negligible differences. In Zone B, this figure grows to around 10%. In Zone C, the value rises to around 15% which, although not negligible, it is still tolerable in any numerical modeling of human events. In Zone D, the difference reaches about 20% thereafter, which is equivalent to the last 10 days, for Brazil, there is indeed a big difference, so to make any extrapolation, care must be taken to avoid using these values, if one uses the database of public notaries. But this phenomenon is only observable in a case as vast as that of the whole of Brazil. See the example of the city of Rio de Janeiro.


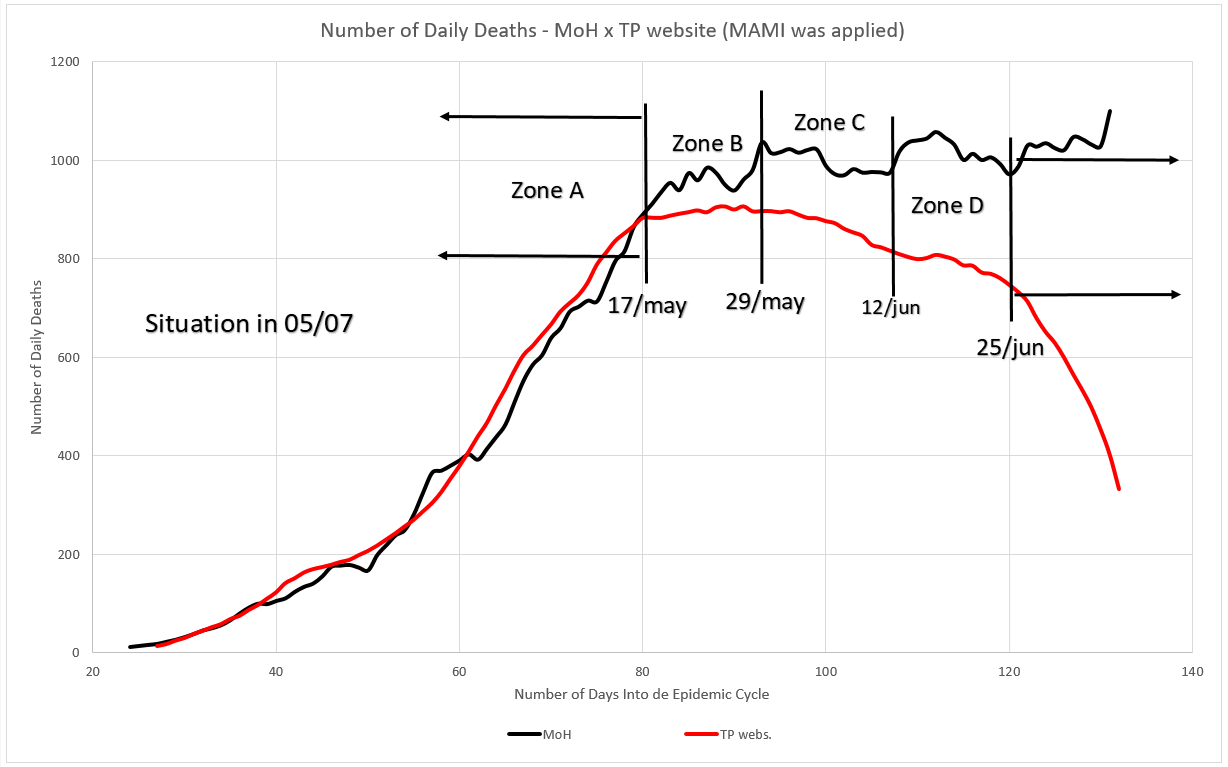


Figure A7 – Number of daily deaths according to MoH (black line) and Transparency Portal (red).
